# Rapid Room‐Temperature Synthesis of Te^4+^‐Doped Cs_2_ZrCl_6_ Vacancy‐Ordered Double Perovskites With Tunable Luminescence Triggered by Extreme Conditions for Advanced Optical Thermometry and Manometry

**DOI:** 10.1002/advs.202523940

**Published:** 2026-02-23

**Authors:** Zhiyu Pei, Yeshan Wu, Shuailing Ma, Tian Cui, Laihui Luo, Peng Du

**Affiliations:** ^1^ School of Physical Science and Technology Ningbo University Ningbo Zhejiang China

**Keywords:** double perovskites, high‐pressure luminescence, optical manometry, optical thermometry

## Abstract

Te^4+^‐doped Cs_2_ZrCl_6_ vacancy‐ordered double perovskites with intense self‐trapped excitons emissions were prepared via a rapid room‐temperature precipitation method. Utilizing low‐temperature engineering, tunable luminescence was realized in the synthesized products, resulting in the maximum temperature relative sensitivities of 0.48% and 0.81% K^−1^, respectively, when the emission band centroid and lifetime were employed as thermometric parameters. Moreover, in situ high‐pressure Raman spectra and X‐ray diffraction patterns confirmed the excellent structural stability and reversibility of the studied samples. When the as‐prepared compounds experienced the high‐pressure conditions, spectral blue‐shift and broadened bandwidth were observed, resulting in the tunable luminescence at high‐pressure, which endowed their applications in pressure sensing. Furthermore, via utilizing the emission band centroid and full width at half maximum as manometric parameters, the maximum pressure sensitivities of the final products were 6.30 and 1.86 nm GPa^−1^, respectively. Additionally, based on the pressure‐related color coordinate, the manometric properties of the resultant products were further investigated, yielding a maximum sensitivity of 4.25% GPa^−1^. Our findings did not only propose a rapid synthesis route for the Te^4+^‐doped Cs_2_ZrCl_6_ vacancy‐ordered double perovskites but also highlighted that their luminescence properties can be regulated via extreme conditions engineering, showcasing their feasibilities in advanced optical thermometry and manometry.

## Graphical abstract

Through utilizing a proposed rapid room‐temperature precipitation method, the Te^4+^‐doped Cs_2_ZrCl_6_ vacancy‐ordered doubles with intense STE emission were synthesized. Via using the extreme conditions, i.e., temperature and pressure, stimulus, the luminescence properties of the resulting compounds are significantly modulated, endowing their promising applications in multi‐parameter optical thermometry and manometry.

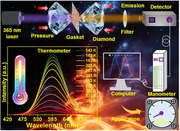

## 1 Introduction

Pressure and temperature, as two extreme conditions, do not only impact the basic characteristics (i.e., material state, phase composition, electronic structure, etc.) of materials but also are crucial to new materials synthesis, industrial manufacture and scientific research [1, 2]. Thus, their accurate monitoring with high sensitivity and high spatial resolution is necessary. Compared with the contacted calibration techniques, the remote luminescence monitoring routes have attracted considerable attentions owing to their admirable features including fast response, contactless detection, excellent resolution and the usable in some harsh environments, such as oil/gas industry, micro or nano‐sized objects, and so forth [3, 4]. As is well documented, through taking advantage of the thermal‐dependent optical spectroscopy properties, i.e., fluorescence intensity, bandwidth, lifetime, etc., of luminescent materials [5, 6, 7], remote optical thermometry is feasible. In comparison, the self‐calibrated ratiometric luminescence thermometers have been intensively investigated, in which the fluorescence intensity ratio of two emissions from rare‐earth or transition metal ions is adopted as the temperature indicator [8, 9]. Although this route presents a strong resistance to the external factors, such as excitation pump power, dopant content (i.e., luminescence intensity), etc., the complex interactions between light and medium will generate inevitable influence on the temperature readout [10]. Consequently, to overcome this shortage, more attention should be paid to analyzing the responses of the other spectroscopy parameters, i.e., lifetime and emission band position, to temperature so as to design new optical thermometers with high accuracy and sensitivity.

For the sake of satisfying the high‐pressure scientific research, the diamond anvil cell (DAC) had been developed to produce the hydrostatic high‐pressure, in which the created pressure is calibrated through monitoring the spectral red‐shift of ruby. However, the commonly used ruby exhibits some inherent issues, i.e., low absolute pressure sensitivity (i.e., d*λ*/d*p* = 0.365 nm GPa^−1^) and quenched luminescence at high‐pressure, which are unable to meet with the demands of the development of science and technology. In order to develop highly sensitive optical manometers, many different types of luminescent materials, such as Ba_3_Lu(BO_3_)_3_:Ce^3+^, Sr_2_[Mg_0.9_Li_0.1_Al_4.9_Si_0.1_N_7_]:Eu^2+^ , La_3_Mg_2_SbO_9_:Mn^4+^, Li_3_Sc_2_(PO_4_)_3_:Cr^3+^, NaY_9_(SiO_4_)_6_O_2_:Mn^2+^, etc. [11, 12, 13, 14, 15], were proposed. Although these compounds exhibited higher sensitivities than ruby, most of them still presented quenched luminescence at high‐pressure as well as the relatively narrow operating range (i.e., *p* ≤ 10 GPa). Thus, further efforts are required to develop new pressure sensitive luminescent materials so as to design high quality optical manometers. Nowadays, the impact of the high‐pressure on the characteristics of metal ions (i.e., Bi^3+^, Te^4+^, Mn^2+^, Cr^3+^, etc.) doped double perovskites has been extensively investigated on account of their unique self‐trapped exciton (STE) emissions and the soft nature features [16, 17, 18, 19, 20]. Yuan et al., reported that the near‐infrared luminescence of the Cr^3+^‐doped Cs_2_AgInCl_6_ double perovskites can be regulated using high‐pressure engineering [21]. When the high‐pressure was applied, Zou et al., found the distinct piezochromism behavior, i.e., emitting color changed from yellow to red, in the Mn^2+^‐doped Cs_2_NaBiCl_6_ double perovskites [22]. Liu et al., demonstrated that the STE emission of Cs_2_TeCl_6_ vacancy‐ordered double perovskites was highly dependent on pressure, resulting in pressure‐triggered polychromatic luminescence [23]. Though analyzing the responses of the luminescence of the Bi^3+^‐doped Cs_2_Ag_0.6_Na_0.4_InCl_6_ double perovskites to pressure, Runowski et al., revealed that the designed compounds were suitable for high sensitive optical manometry, i.e., d*λ*/d*p* = 112 nm GPa^−1^ [24]. Inspired by these, the utilization of the metal ions doped double perovskites would be a promising strategy to develop high‐performance optical manometers.

**FIGURE 1:**
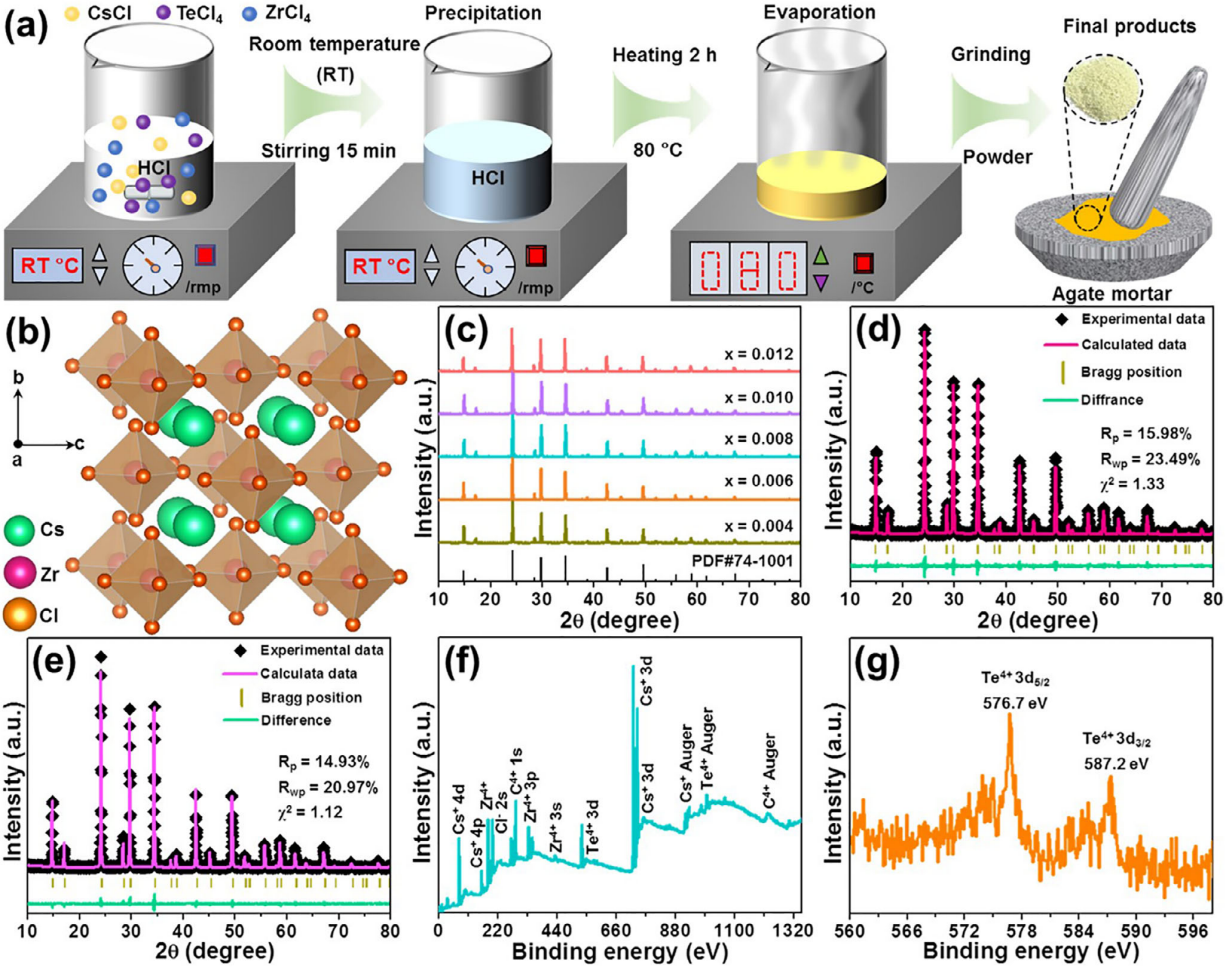
(a) Schematic diagram of the rapid synthesis of the Te^4+^‐doped Cs_2_ZrCl_6_ vacancy‐ordered double perovskites at room‐temperature. (b) Crystal structure of the Cs_2_ZrCl_6_ unit cell. (c) XRD profiles of the Cs_2_ZrCl_6_:*x*Te^4+^ vacancy‐ordered double perovskites. Rietveld XRD refinement results of the studied samples with the Te^4+^ content of (d) *x* = 0.008 and (e) *x* = 0.012. (f) Full XPS spectrum of the Cs_2_ZrCl_6_:0.008Te^4+^ vacancy‐ordered double perovskites and (g) High‐resolution XPS spectrum of Te^4+^ 3d in the prepared double perovskites.

Recently, considerable attention has been gained in the metal ions doped vacancy‐ordered double perovskites to develop novel luminescent materials, such as Cs_2_ZrCl_6_:Bi^3+^, Cs_2_ZrCl_6_:Te^4+^, Cs_2_ZrCl_6_:Sb^3+^, Rb_2_ZrCl_6_:Te^4+^, and so on [25, 26, 27, 28, 29, 30]. In comparison, the Te^4+^‐doped Cs_2_ZrCl_6_ vacancy‐ordered double perovskites with intense yellow emission have been widely studied for white light‐emitting diode applications [31, 32, 33, 34, 35]. Note that, to prepare these designed compounds, the hydrothermal method had been adopted, whereas it suffered from complex operation process, long reaction time, small products etc., limiting its further development. Furthermore, to the best of our knowledge, the reports on the luminescence and crystal structure of the metal ions doped Cs_2_ZrCl_6_ vacancy‐ordered double perovskites at high‐pressure conditions are limited, let alone their feasibilities in optical manometry. In this work, to settle these shortages, the Te^4+^ and Cs_2_ZrCl_6_ were selected as dopant and host, respectively, to synthesize the Cs_2_Zr_1‐_ *_x_* Cl_6_:*x*Te^4+^ (Cs_2_ZrCl_6_:*x*Te^4+^) vacancy‐ordered double perovskites. Herein, we proposed a facile rapid room‐temperature precipitation method to prepare the designed compounds and the corresponding synthetic processes are described in Figure 1a. The phase composition, elemental composition, morphology and luminescence properties of the resulting double perovskites were systematically investigated. According to the responses of the emission band centroid and lifetime to temperature, the thermometric behaviors of the prepared double perovskites were determined, implying their feasibilities in low‐temperature monitoring. Moreover, via using the in situ absorption spectra, Raman spectra and X‐ray diffraction (XRD) profiles, the evolution of the optical band gap and phase structure of the studied samples had been explored. Furthermore, as pressure elevates, both spectral blue‐shift and narrowed bandwidth were simultaneously observed in the final products, endowing their applications in optical manometry. Additionally, utilizing the pressure‐related emission band centroid, bandwidth and color coordinates as manometric parameters, multi‐parameter visual optical manometers with high sensitivity and broad operating range were developed. Our findings suggested that luminescence features of the Te^4+^‐doped Cs_2_ZrCl_6_ vacancy‐ordered double perovskites can be efficiently manipulated through extreme conditions, enabling their applications in advanced optical thermometry and manometry.

## 2 Results and Discussion

For the crystal structure of the Cs_2_ZrCl_6_ unit cell, the Zr atom is surrounded by six Cl atoms, forming an octahedral structure, while the Cs atom is coordinated to twelve Cl atoms, resulting in a tetradecahedral structure (Figure 1b). The XRD profiles of the Cs_2_ZrCl_6_:*x*Te^4+^ vacancy‐ordered double perovskites are shown in Figure 1c. As disclosed, the resulting compounds exhibit similar diffraction patterns and they all coincide well with the standard cubic Cs_2_ZrCl_6_ (PDF#74‐1001), demonstrating that the doping of Te^4+^ can not alter the phase structure of the studied samples. Moreover, to get deeper insight into the phase compositions of the designed products, Rietveld XRD refinements of the Cs_2_ZrCl_6_:0.008Te^4+^ and Cs_2_ZrCl_6_:0.012Te^4+^ vacancy‐ordered double perovskites were conducted, as descried in the Figure 1d,e, respectively. Apparently, these calculated diffraction profiles are in accordance well with the experimental data, further implying that the resultant vacancy‐ordered double perovskites have pure cubic phase with *Fm‐3m* space group. Note that, owing to the different ionic radii between Te^4+^ and Zr^4+^, the lattice parameters, i.e., *a* = *b* = *c* and volume, are boosted with the increment of Te^4+^ content (Table S1). On the other hand, for the purpose of exploring the elemental compositions as well as their valence states in the resulting samples, the X‐ray photoelectron spectroscopy (XPS) measurement was carried out. As shown in the full XPS spectrum (Figure 1f), the prepared compounds contain the Cs, Zr, Cl and Te elements. Particularly, two bands with the binding energies of 724.2 and 738.2 eV, which are attributed to the Cs^+^ 3d_5/2_ and Cs^+^ 3d_3/2_, respectively, are observed in the high‐resolution XPS spectrum of Cs^+^ 3d (Figure S1a) [36], and the high‐resolution XPS spectrum of Zr^4+^ 3d also contains two peaks at 182.8 and 185.3 eV corresponding to Zr^4+^ 3d_5/2_ and Zr^4+^ 3d_3/2_, respectively, as presented in Figure S1b [37]. Furthermore, the high‐resolution XPS spectrum of Cl^−^ 2p can be deconvoluted into two peaks at 198.6 and 200.2 eV, which are assigned to Cl^−^ 2p_3/2_ and Cl^−^ 2p_1/2_, respectively (see Figure S1c) [36]. Additionally, the existence of Te^4+^ in the developed double perovskites is verified by the high‐resolution XPS spectrum (Figure 1g), of which two bands with the binding energies of 576.7 and 587.2 eV pertaining to the Te^4+^ 3d_5/2_ and Te^4+^ 3d_3/2_, respectively, are detected [37]. These results reveal that the Te^4+^‐doped Cs_2_ZrCl_6_ vacancy‐ordered double perovskites are successfully synthesized via a facile room‐temperature precipitation method.

**FIGURE 2:**
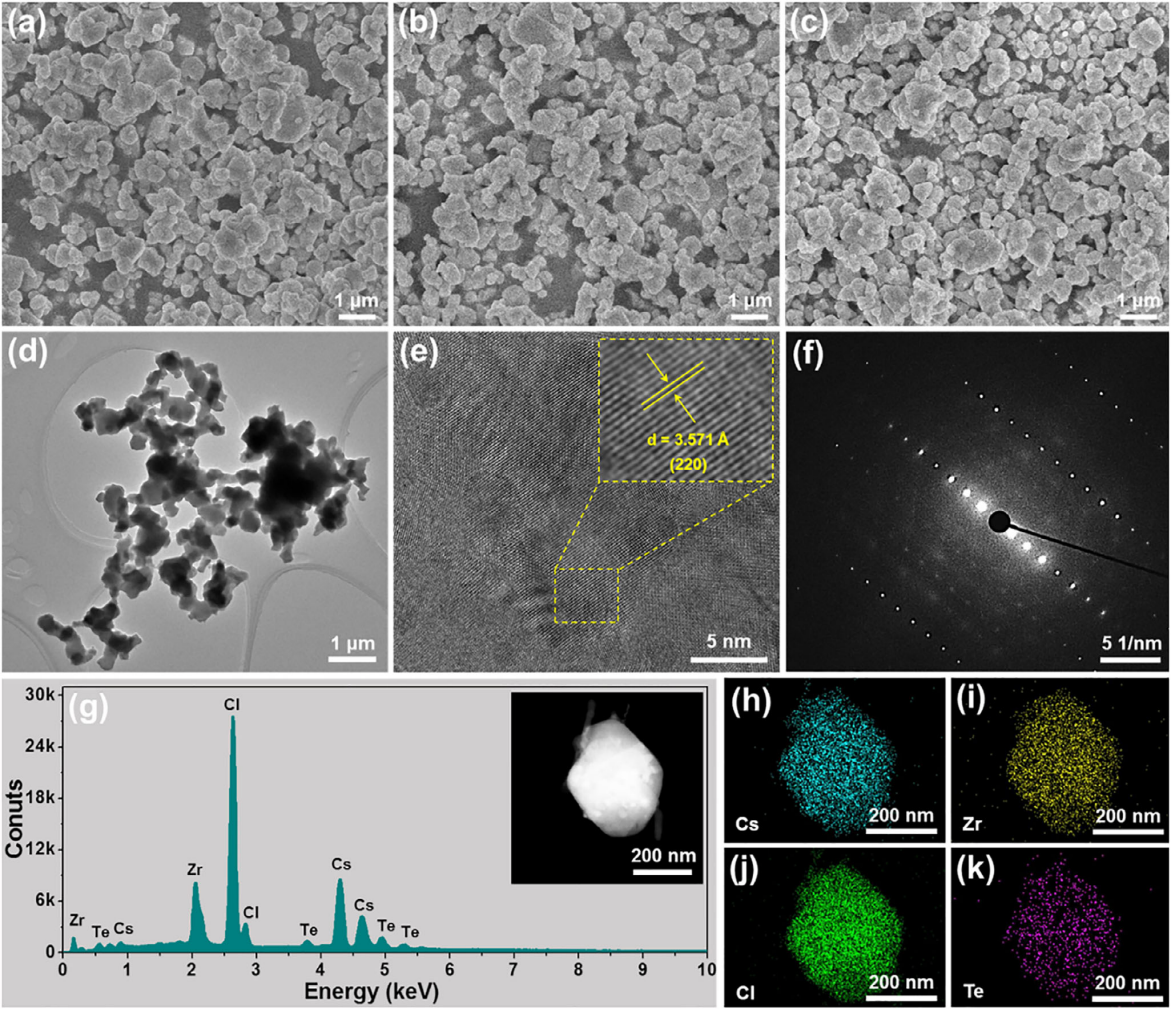
SEM graphs of the Cs_2_ZrCl_6_:*x*Te^4+^ vacancy‐ordered double perovskites with the Te^4+^ contents of (a) *x* = 0.004, (b) *x* = 0.008 and (c) *x* = 0.012. (d) TEM image, (e) High‐resolution TEM image, (f) SAED pattern, (g) EDS spectrum and (h)‐(k) Elemental results of the Cs_2_ZrCl_6_:0.008Te^4+^ vacancy‐ordered double perovskites. Insest of (g) shows the SEM image used for elemental mapping measurements.

The impact of Te^4+^ doping on the morphological characteristics of the resulting compounds is explored via using the scanning electron microscopy (SEM) and transmission electron microscope (TEM). From the recorded SEM images (Figure 2a‐c; Figure S2), one knows that the synthesized vacancy‐ordered double perovskites consist of inhomogeneous particles, in which their particle size and shape are independent on the Te^4+^ content. Moreover, the irregular particles in the studied samples are also confirmed through the TEM image (see Figure 2d). It is demonstrated in Figure 2e that the high‐resolution TEM image of the resulting vacancy‐ordered double perovskites are composed of distinct lattice fringes and their adjacent spacing is 3.571 Å, which is attributed to the (220) crystal plane of the Cs_2_ZrCl_6_ vacancy‐ordered double perovskites. Furthermore, lots of bright diffraction spots are gained in the selected‐area electron diffraction (SAED) pattern, as shown in Figure 2f, manifesting the single‐crystal features of the designed compounds. Additionally, the energy‐dispersive X‐ray spectroscopy (EDS) result shown in Figure 2g also proves that the final products contain the elements of Cs, Zr, Cl and Te, of which they are evenly distributed throughout the entire particles, as verified by the elemental mapping results (see Figure 2h‐k).

**FIGURE 3:**
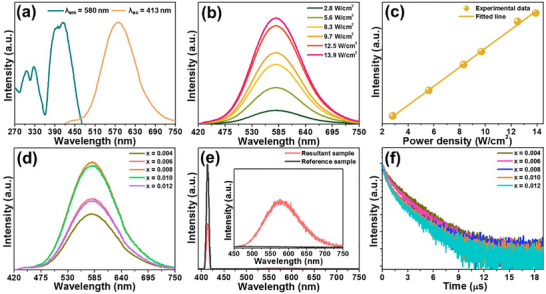
(a) Normalized excitation and emission spectra of the Cs_2_ZrCl_6_:0.008Te^4+^ vacancy‐ordered double perovskites. Pump power dependent (b) emission spectra and (c) fluorescence intensity of the Cs_2_ZrCl_6_:0.008Te^4+^ double perovskites. (d) Emission spectra of the Cs_2_ZrCl_6_:*x*Te^4+^ vacancy‐ordered double perovskites. (e) Quantum efficiency test of the Cs_2_ZrCl_6_:0.008Te^4+^ double perovskites excited at 413 nm. (f) Decay curves of the Cs_2_ZrCl_6_:*x*Te^4+^ vacancy‐ordered double perovskites.

The normalized excitation and emission spectra of the Cs_2_ZrCl_6_:0.008Te^4+^ vacancy‐ordered double perovskites are shown in Figure 3a. Monitored at 580 nm, the recorded excitation spectrum includes two broad bands, i.e., the shorter one, spanning 270–360 nm, originates from the ^1^S_0_ → ^3^P_2_ transition of Te^4+^, and the longer one, ranging from 360 to 470 nm, arises from the ^1^S_0_ → ^3^P_1_ transition of Te^4+^ [23, 32]. Under excitation at 413 nm, the resulting products exhibit a broad emission band at 580 nm with a large Stokes shift of 167 nm (see Figure 3a). For the sake of exploring the origin of the generated emission, the excitation power density dependent emission spectra were tested and presented in Figure 3b. Evidently, the fluorescence intensity is linearly boosted as excitation power density arises (Figure 3c), suggesting that the emission can not be assigned to the permanent defect since its emission is limited and saturates at high excitation power [37]. Moreover, the room‐temperature lifetime of the emission at 580 nm in the Cs_2_ZrCl_6_:0.008Te^4+^ vacancy‐ordered double perovskites is found to be 1.54 µs (see Figure S3a), excluding the free exciton emission since its lifetime is in nanosecond scale [38]. In view of the large Stokes shift, long lifetime and unsaturated emission intensity, the observed broad emission band originates from the recombination of STE. Ultimately, the energy level diagram of the host and Te^4+^ as well as the possible luminescence process is described in Figure S3b.

To explore the optimal doping content, the emission spectra of the Cs_2_ZrCl_6_:*x*Te^4+^ vacancy‐ordered double perovskites were examined and presented in Figure 3d. As disclosed, the broad emission bands are seen in every sample, of which their emission band positions are hardly impacted by the dopant content, while the fluorescence intensity shows different change tendency. Specifically, as Te^4+^ content arises, the fluorescence intensity is enhanced and achieves its strongest state at *x* = 0.008, in which the concentration quenching arises when the dopant content exceeds 0.8 mol%, as presented in Figure S4a. Herein, the critical distance (*R_c_*) of Te^4+^ in Cs_2_ZrCl_6_ host lattices is estimated to be 40.8 Å (i.e., larger than 5 Å) (for calculation detail, see Supporting Information), suggesting that the concentration quenching results from the electric dipolar interaction. Moreover, according to the relation between fluorescence intensity and doping content, the involved concentration quenching mechanism is further confirmed to be electric dipole‐dipole interaction, as displayed in Figure S4b. Furthermore, irradiated by NUV light, it is found that the designed vacancy‐ordered double perovsiktes can emit dazzling yellow emission with color coordinate of (0.467,0.498) (see Figure S4c). In addition, upon the excitation at 413 nm, the internal quantum efficiency of the Cs_2_ZrCl_6_:0.008Te^4+^ vacancy‐ordered double perovskites is determined to be 31.9%, as presented in Figure 3e. These results suggest that the Te^4+^‐doped Cs_2_ZrCl_6_ vacancy‐ordered double perovskites possess good luminescence characteristics, enabling their vivid applications in various fields.

As stated above, the Te^4+^‐doped Cs_2_ZrCl_6_ vacancy‐ordered double perovskites are able to be prepared via a rapid room‐temperature synthetic route (i.e., reaction time is 15 min). So can the characteristics, i.e., phase composition, morphology and luminescence, of the studied samples can impacted by the preparation conditions, such as reaction time and temperature? In order to settle these doubts, we prepared the Cs_2_ZrCl_6_:0.008Te^4+^ vacancy‐ordered double perovskites under different reaction time and temperature, with all synthetic processes consistent with those presented in Figure 1a. It is shown in Figure S5 that the change of the reaction temperature (i.e., from 30°C to 90°C) and time (i.e., 15–120 min) is unable to tune the phase structure of the designed compounds. Notably, with rising the reaction temperature, it is found that the morphological features of the synthesized compounds are almost unchanged, as displayed in Figure S6. In comparison, when the reaction time increases from 15 to 120 min, it can be seen that the particle sizes of the prepared samples are increased, as depicted in Figure S7. Furthermore, excited by 413 nm, it is clear all of these samples can exhibit the intense STE emissions and their fluorescence intensities are mildly impacted by the reaction temperature and time, as demonstrated in Figure S8. These features reveal that our proposed room‐temperature preparation route is an efficient and facile strategy to synthesize the Te^4+^‐doped Cs_2_ZrCl_6_ vacancy‐ordered double perovskites.

In an attempt to study the luminescence dynamic process in the designed compounds, the room‐temperature decay curves of the Cs_2_ZrCl_6_:*x*Te^4+^ vacancy‐ordered double perovskites were detected and depicted in Figure 3h, of which the excitation and monitoring wavelengths are 413 and 580 nm, respectively. Here, these recorded decay curves are all able to be fitted via a double exponential expression, as defined below:

(1) $$I\;\left(t\right)=A_{1}\;\exp\left(-t/\tau_{1}\right)+A_{2}\exp\left(-t/\tau_{2}\right)$$

where the fluorescence intensity at time *t* is labeled by I(*t*), *A_i_* (*i* = 1, 2) denotes the constants, and the lifetime is labeled by *τ_i_* (*i* = 1, 2). Besides, with the aid of the following formula, the average lifetime (*τ*) is estimated:

(2) $$\tau =\frac{A_{1}\tau_{1}^{2}+A_{2}\tau_{2}^{2}}{A_{1}\tau_{1}+A_{2}\tau_{2}}\;$$

Accordingly, the lifetimes of the emission in the Cs_2_ZrCl_6_:*x*Te^4+^ vacancy‐ordered double perovskites are 1.94, 1.74, 1.54, 1.49 and 1.39 µs, respectively, when *x* = 0.004, 0.006, 0.008, 0.010 and 0.012. It is significant that the decay time decreases as doping content arises, further manifesting that the concentration quenching happens in the resulting samples.

**FIGURE 4:**
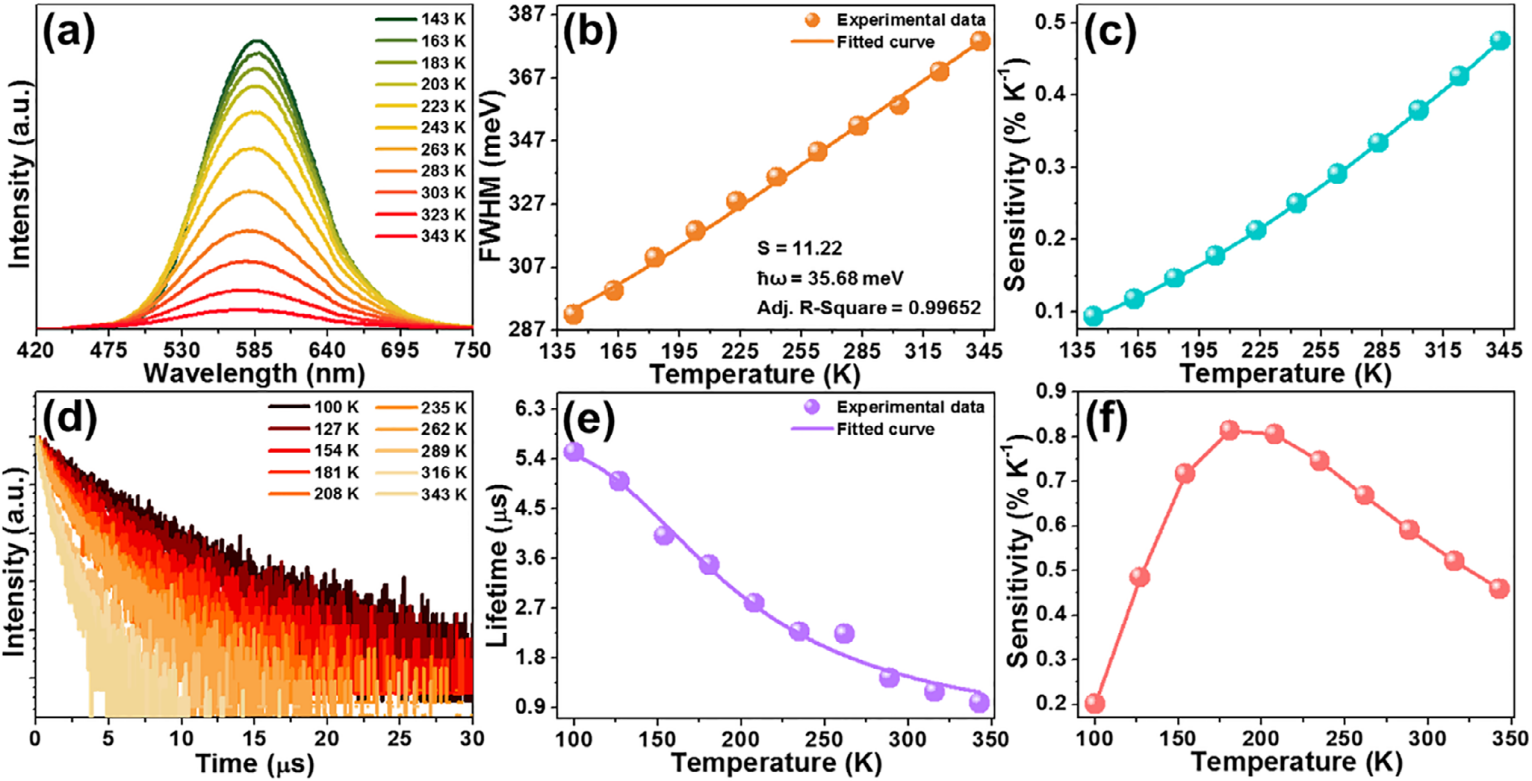
Temperature‐dependent (a) emission spectra, (b) FWHM and (c) sensitivity of the Cs_2_ZrCl_6_:0.008Te^4+^ vacancy‐ordered double perovskites. (d) Decay curves, (e) lifetime and (f) sensitivity of the Cs_2_ZrCl_6_:0.008Te^4+^ vacancy‐ordered double perovskites as a function of temperature.

To analyze the STE dynamics and exciton‐phonon in the designed compounds, the temperature‐dependent emission spectra of the Cs_2_ZrCl_6_:0.008Te^4+^ vacancy‐ordered double perovskites in the range of 143–343 K were recorded and illustrated in Figure 4a. As temperature elevates, the STE emission intensity decreases monotonously owing to the thermally‐associated nonradiative recombination, endowing its feasibility in optical temperature sensing. For a more in‐depth analysis, the activation energy (*E_a_*) is estimated via using the following equation: [39, 40]

(3) $$I\;\left(T\right)=\frac{I_{0}}{1+A\exp\left(-E_{a}/k_{B}T\right)}\;$$

where the integrated fluorescence intensities at temperature *T* and *T* = 0 K are represented by I(*T*) and I_0_, respectively, *A* is constant and *k_B_* stands for the Boltzmann constant. Consequently, the *E_a_* value of the Cs_2_ZrCl_6_:0.008Te^4+^ vacancy‐ordered double perovskites is decided to be 170 meV (see Figure S9). Note that, despite thermally quenched luminescence, the STE emission band broadens at high temperature, i.e., its full width at half maximum (FWHM) increases from 291.9 to 378.7 meV, which is assigned to the increased electron‐phonon interaction, as the temperature rises from 143 to 343 K (Figure 4b). As is well documented, to form STE, a strong electron‐phonon coupling is required and it can be evaluated through utilizing the Huang‐Rhys factor (i.e., *S*), of which the *S* value is able to be determined by studying the temperature‐dependent FWHM using the following function: [40, 41]

(4) $$\mathrm{FWHM}\;=2.36\sqrt{S}\hbar w\sqrt{\coth\left(\frac{\hbar \omega_{phonon}}{2k_{B}T}\right)}$$

where *ħω_phonon_* describes the phonon energy. From Figure 4b, one knows that the *S* and *ħw* of the resulting double perovskites are 11.22 and 35.68 meV, respectively. Since the calculated *S* value is larger than 5, it is reasonable to consider that the designed compounds possess strong exciton‐phonon coupling [42], which is beneficial for the production of more STE, contributing to the intense STE emission with large Stokes shift. On the other hand, the temperature‐triggered spectral blue‐shift is also observed in the studied samples, as verified by the normalized temperature‐dependent emission spectra (see Figure S10a). Specifically, as temperature rises (i.e., 143–343 K), it is found that the emission band centroid (*λ*) changes from 583.8 to 572.4 nm (Figure S10b), in which the relation between temperature and emission band centroid can be expressed through using a third‐order polynomial function, namely, *λ* = 1.027 × 10^−6^ *T* ^3^ – 8.759×10^−4^ *T* ^2^ + 0.176*T* + 573.494. To evaluate the thermometric properties of the developed products, the relative temperature sensitivity, *S_R_* (*T*), is investigated, as follows [37]:

(5) $$S_{R}\;\left(T\right)=\left|\frac{1}{\lambda }\frac{d\lambda }{dT}\right|\;\times 100\%$$

The calculated *S_R_* (*T*) values are presented in Figure 4c. As disclosed, a gradual enhancement is seen in the *S_R_* (T) value, reaching its maximum value of 0.48% K^−1^ at 343 K. This feature indicates that the Te^4+^‐doped Cs_2_ZrCl_6_ vacancy‐ordered double perovskites possess the capability of monitoring low‐temperature using the emission band centroid as a temperature indicator.

For the purpose of further investigating the thermometric properties of the studied samples, the temperature‐related decay curves of the Cs_2_ZrCl_6_:0.008Te^4+^ vacancy‐ordered double perovskites in the range of 100–343 K were examined and shown in Figure 4d. Through analyzing the recorded decay curves, one knows that the lifetime presents a downward tendency as temperature elevates (i.e., 100–343 K), namely, it alters from 5.52 to 0.99 µs (see Figure 4e). As reported previously, the lifetime and temperature satisfy the following relationship [42, 43]:

(6) $$\tau \;\left(T\right)=\frac{\tau_{0}}{1+\exp\left(-\Delta E/k_{B}T\right)}\;$$

where *τ*(*T*) and *τ*_0_ are the lifetime at the measured temperature *T* and *T* = 0 K, respectively, and Δ*E* is the activation energy. Though utilizing the aforementioned formula to analyze the experimental data, we found that the relationship between lifetime and temperature can be described as: *τ =* 5.63/[1 + 27.77exp(−681.68/*T*)]. Furthermore, via using the following expression, the temperature‐associated *S_R_* (*T*) value is determined [42, 43]:

(7) $$S_{R}\;\left(T\right)=\left|\frac{1}{\tau }\frac{d\tau }{dT}\right|\;\times 100\%$$

The calculated *S_R_* (*T*) value is depicted in Figure 4f. As temperature increases, it is significant that the *S_R_* (*T*) value exhibits an upward tendency, presenting the maximum value of 0.81% K^−1^ at 208 K. This result demonstrates that the Te^4+^‐doped Cs_2_ZrCl_6_ vacancy‐ordered double perovskites are suitable for temperature measurement in the cryogenic range, utilizing lifetime as the thermometric parameter.

**FIGURE 5:**
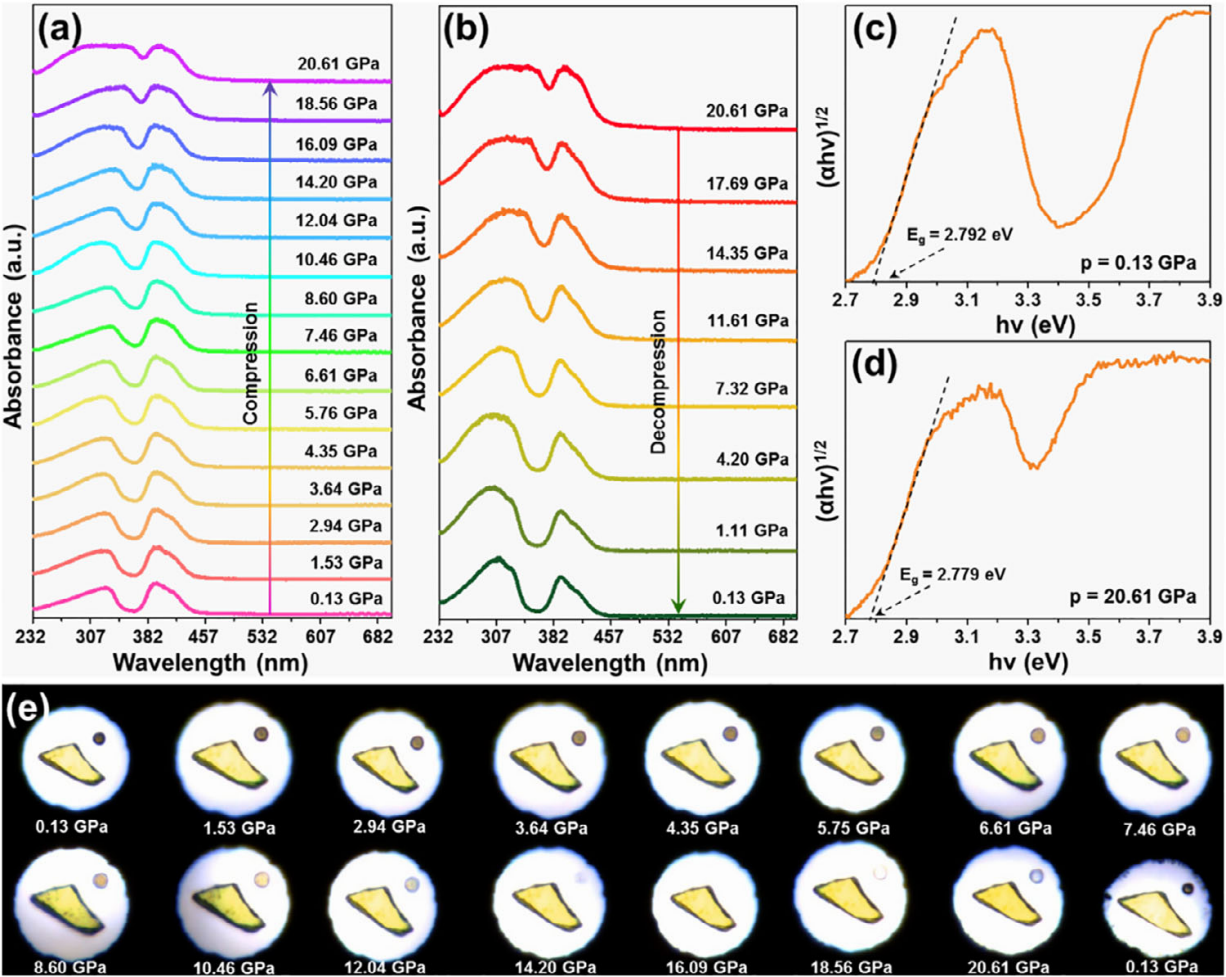
In situ high‐pressure absorption spectra of the Cs_2_ZrCl_6_:0.008Te^4+^ vacancy‐ordered double perovskites during (a) compression and (b) decompression process. Plots of the (*αhv*)^1/2^ *vs*. *hv* for the Cs_2_ZrCl_6_:0.008Te^4+^ vacancy‐ordered double perovskites at (c) *p* = 0.13 GPa and (d) *p* = 20.61 GPa. (e) Optical micrographs of the Cs_2_ZrCl_6_:0.008Te^4+^ vacancy‐ordered double perovskites at high‐pressure.

To investigate the impact of pressure on the light harvest ability of synthesized products, their in situ high‐pressure absorption spectra were tested. Figure 5a presents the pressure‐dependent absorption spectra of the Cs_2_ZrCl_6_:0.008Te^4+^ vacancy‐ordered double perovskites during the compression process (i.e., 0.13–20.61 GPa). As demonstrated, two distinct absorption peaks, of which the shorter wavelength originates from the ^1^S_0_ → ^3^P_2_ transition of Te^4+^, and the longer wavelength arises from the ^1^S_0_ → ^3^P_1_ transition of Te^4+^, are observed in these absorption spectra [23]. As pressure rises, the absorption peaks undergo a gradual red‐shift due to the homogenous decrease in octahedral volume [44], resulting in the pressure‐caused optical band gap evolution. Notably, when the studied samples experience the decompression process (i.e., releases the pressure), the absorption bands can revert to their initial states, as presented in Figure 5b, suggesting the good reversibility of pressure‐induced optical band gap evolution. For the sake of better describing the optical band gap evolution at high‐pressure, its value is estimated by means of the following function [45]:

(8) $$\alpha hv=A{\left(hv-E_{g}\right)}^{n}$$

where *hv* stands for the photon energy, *A* is constant, *α* denotes the absorption coefficient, *E_g_* is the optical band gap and *n* value is decided by the semiconductor category. Since Cs_2_ZrCl_6_ pertains to the indirect semiconductor [46, 47], the *n* value is 2. The typical plots of (*αhv*)^1/2^ *vs*. *hv* for the Cs_2_ZrCl_6_:0.008Te^4+^ vacancy‐ordered double perovskites at the pressure of 0.13 and 20.61 GPa are presented in Figure 5c,d, respectively. As demonstrated, when *p* = 0.13 GPa, the *E_g_* value of the Cs_2_ZrCl_6_:0.008Te^4+^ vacancy‐ordered double perovskites is 2.792 eV, while it decreases to 2.779 eV when the pressure increases to 20.61 GPa. As is well documented, the bond length will be reduced at high‐pressure, which is conducive to promoting the electronic orbital overlap, leading to the contraction of the optical band gap [23]. Figure 5e depicts the piezochromic transition of the prepared vacancy‐ordered double perovskites during the compression‐decompression processes. With elevating the pressure (i.e., 0.13–20.61 GPa), a piezochromism behavior takes place, i.e., from pale yellow to yellow, further clarifying that the optical band gap is able to be regulated by high‐pressure engineering. Additionally, when the pressure releases to 0.13 GPa, both the *E_g_* value and color of the vacancy‐ordered double perovskites can return to its starting states (see Figure 5e; Figure S11).

**FIGURE 6:**
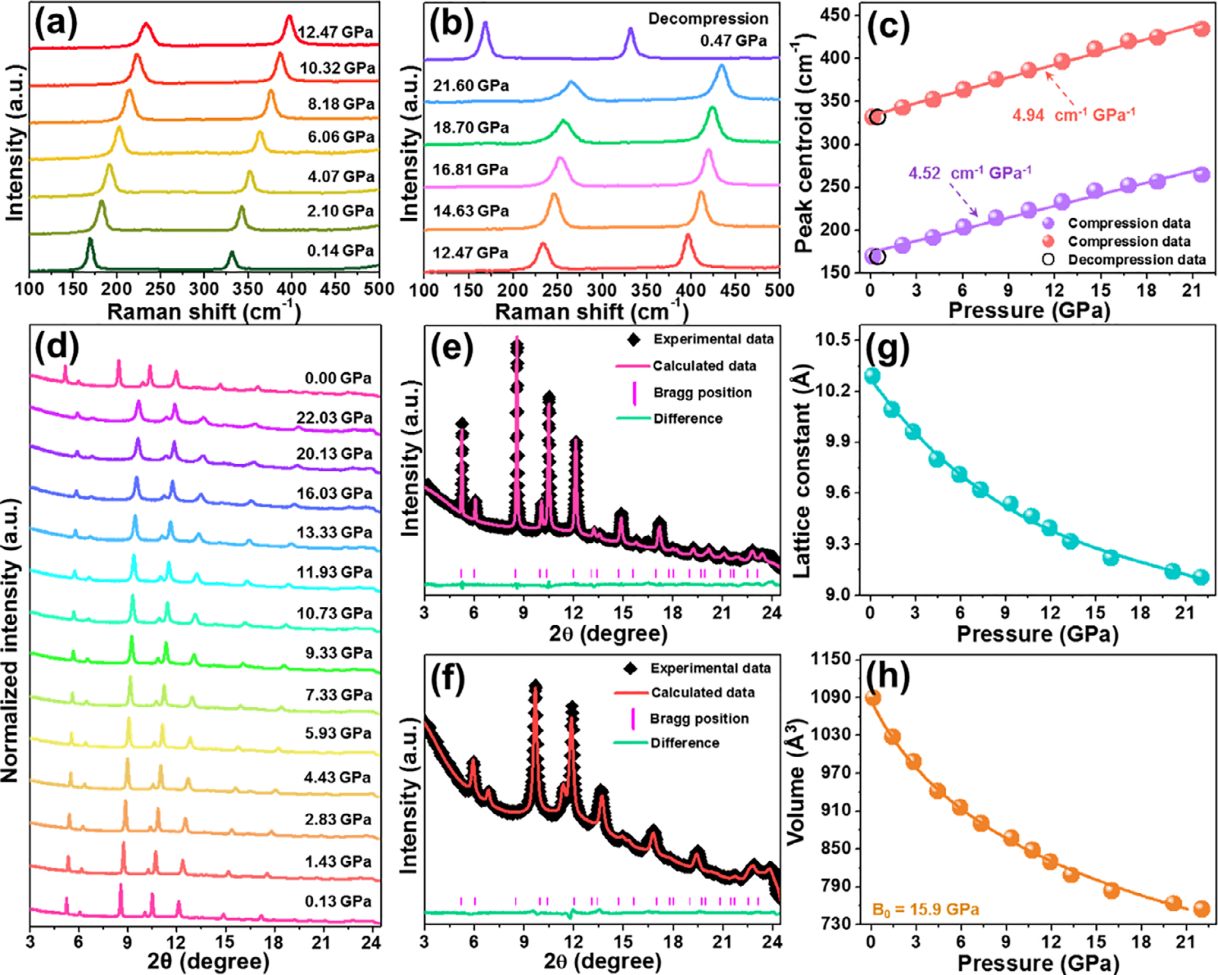
(a) and (b) In situ high‐pressure Raman spectra of the Cs_2_ZrCl_6_ vacancy‐ordered double perovskites in the range of 0.14–21.60 GPa. (c) Determined Raman peak centroids as a function of pressure. (d) In situ high‐pressure XRD profiles of the Cs_2_ZrCl_6_:0.008Te^4+^ vacancy‐ordered double perovskites. Rietveld XRD refinements of the Cs_2_ZrCl_6_:0.008Te^4+^ vacancy‐ordered double perovskites recorded at (e) *p* = 0.13 and (f) *p* = 22.03 GPa. Pressure‐dependent (g) lattice parameter (i.e., *a* = *b* = *c*) and (h) volume of the Cs_2_ZrCl_6_:0.008Te^4+^ vacancy‐ordered double perovskites.

To explore the evolution of the phase structure under compression, the pressure‐related Raman spectra of the Cs_2_ZrCl_6_ vacancy‐ordered double perovskites were measured. As disclosed, the Raman spectrum initially comprises two bands at around 169.1 and 331.8 cm^−1^, which are attributed to the breath vibration of Zr─Cl bond (T_2g_) and symmetric stretching (A_1g_) of [ZrCl_6_]^2−^ octahedron, respectively [36]. Evidently, during the compression process (i.e., 0.14–21.60 GPa), these Raman peaks presents a migration to high wavenumbers, without generating any new peaks from impurity phase, as displayed in Figure 6a,b, demonstrating that the designed vacancy‐ordered double perovskites possess splendid structural stability in the pressure range of our interest. The shortened bond length triggered by high‐pressure can be responsible for the shift of the Raman bands. Moreover, as pressure decreases (i.e., decompression process), one knows that the Raman peaks are able to revert to their initial status (i.e., shifting to low wavenumbers), as shown in Figure 6b, manifesting that the studied samples own good structural reversibility. Furthermore, it is demonstrated in Figure 6c that the relationship between pressure and Raman peak centroids is linear, of which the shift rates of the T_2g_ and A_1g_ Raman modes are 4.52 and 4.94 cm^−1^ GPa^−1^, respectively. Note that, apart from the linear shift, the Raman bands are also gradually broadened at elevated pressure, which results from the promoted amount of crystal defects and strains in the contracted compounds. These characteristics indicate that the designed vacancy‐ordered double perovskites possess a stable phase structure within the pressure range of 0.14–21.60 GPa.

In order to further study the structural stability of the prepared compounds, in situ high‐pressure XRD measurements of the Cs_2_ZrCl_6_:0.008Te^4+^ vacancy‐ordered double perovskites were conducted and the corresponding results are illustrated in Figure 3d. Since the high irradiation energy (*λ* = 0.5427 Å) was employed to measure the pressure‐associated XRD, the diffraction peak positions recorded under high pressure differ from those of the XRD data measured at ambient conditions. During the compression process (i.e., 0.13–22.03 GPa), it can be seen from Figure 3d that neither the diffraction peaks are disappeared nor new diffraction peaks from secondary phase are formed, stating that the studied samples maintain the pure cubic phase within the applied pressure range, which ensures its applications in high‐pressure environments. Nevertheless, all diffraction peaks do not only shift to the higher angles but also are broadened at high‐pressure (see Figure 3d), contributing to the lattice contraction. As is known, the pressure‐induced strains, together with crystal defects, are responsible for the broadening of diffraction peaks. Furthermore, when the pressure releases, it is evident that all diffraction peaks can revert to their original positions (Figure 3d), demonstrating that the developed vacancy‐ordered double perovskites have excellent structural reversibility at high‐pressure, which is an extremely important characteristic to meet the requirements of optical sensing applications under high‐pressure conditions. For the sake of getting deeper insight into the influence of pressure on the crystal structure, the Rietveld XRD refinements were carried out and the corresponding results are illustrated in Figure 6e,f and Figure S12. Apparently, the Rietveld refinements of the XRD patterns at different pressures coincide well with the experimental data, endowing us to consider that the Cs_2_ZrCl_6_:0.008Te^4+^ vacancy‐ordered double perovskites present stable phase structure upon compression. Moreover, with raising the pressure, it is found that the lattice parameter (i.e., *a* = *b* = *c*) and unit cell volume decrease gradually, as described in Figure 6g,h, respectively. In particular, the unit cell volume declines from 1089.441 to 754.797 Å^3^ throughout the entire compression process (i.e., 0.13–22.03 GPa). Additionally, via utilizing the third‐order Birch‐Murnaghan formula, the experimental pressure‐dependent unit cell volume can be further analyzed, as defined below [48, 49]:

(9) $$\begin{array}{ccc} p & = & \frac{3B_{0}}{2}\;\left[{\left(\frac{V_{0}}{V}\right)}^{7/3}-{\left(\frac{V_{0}}{V}\right)}^{5/3}\right]\\ & & \times \left\{1+\frac{3}{4}\left(B_{0}^{\prime }-4\right)\left[{\left(\frac{V_{0}}{V}\right)}^{2/3}-1\right]\right\} \end{array}$$

where *V* and *V*_0_ are assigned to the unit cell volume at pressure *p* and *p* = 0 GPa, respectively, *B*_0_ refers to the bulk modulus at ambient condition and *B*′_0_ describes the parameter for the pressure derivative. Accordingly, the *B*_0_ value is determined to be 15.9 GPa, which is smaller than other compounds, such as Cs_2_AgBiBr_6_ (*B*_0_ = 26.6 GPa) and Cs_3_Bi_2_Br_9_ (*B*_0_ = 18.3 GPa) [48, 49], suggesting the soft nature characteristic of the Te^4+^‐doped Cs_2_ZrCl_6_ vacancy‐ordered double perovskites. As a consequence, the utilization of pressure engineering is an efficient route to regulate the local structure of the designed vacancy‐ordered double perovskites, contributing to the pressure‐dependent luminescence.

**FIGURE 7:**
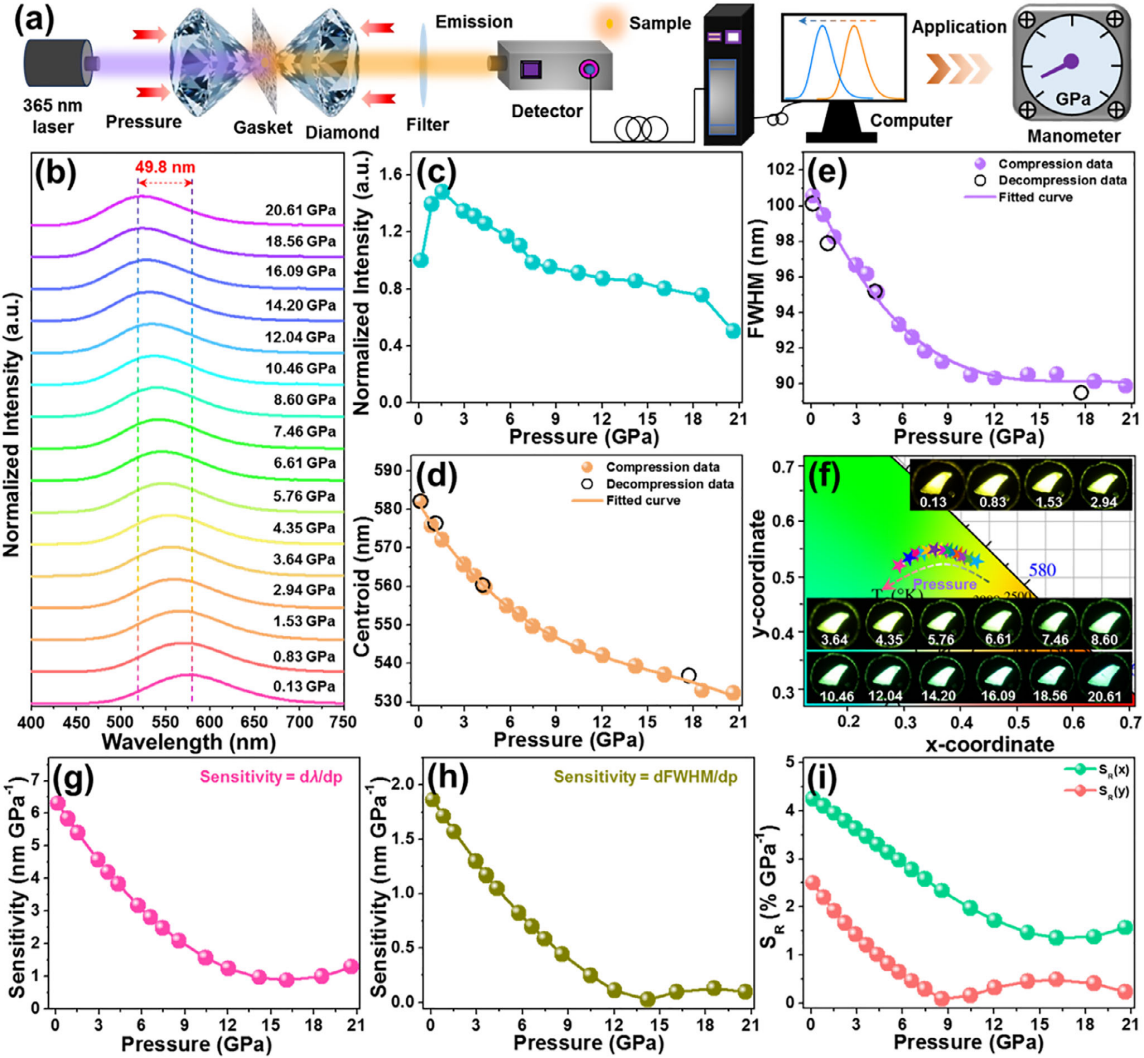
(a) Schematic diagram of the luminescence measurement setup at high‐pressure. Pressure‐associated (b) emission spectra, (c) fluorescence intensity, (d) emission band centroid, (e) FWHM, (f) color coordinate, (g) sensitivity, i.e., d*λ*/d*p* and (h) sensitivity, i.e., dFWHM/d*p* and (i) *S_R_* value of the Cs_2_ZrCl_6_:0.008Te^4+^ vacancy‐ordered double perovskites.

With the aim of investigating the influence of pressure on the luminescence properties of the studied samples, their in situ high‐pressure luminescence properties were characterized and the corresponding experimental setup is described in Figure 7a. The pressure‐dependent emission spectra of the Cs_2_ZrCl_6_:0.008Te^4+^ vacancy‐ordered double perovskites in the range of 0.13–20.61 GPa are presented in Figure 7b and Figure S13a. As demonstrated, with an increase in pressure, the STE emission of the Cs_2_ZrCl_6_:0.008Te^4+^ vacancy‐ordered double perovskites exhibit a distinct spectral blue‐shift accompanied by a changed fluorescence intensity. As pressure rises, the fluorescence intensity increases gradually, achieving its strongest state at *p* = 1.53 GPa (i.e., 1.48 times higher than its initial value), whereas it starts to decreases when *p* ≥ 2.94 GPa, as presented in Figure 7c and Figure S13b. Interestingly, even when the pressure up to 20.61 GPa, the luminescence still does not vanish and it keeps 50.5% of its initial value (see Figure 7c), implying that luminescence of the Cs_2_ZrCl_6_:0.008Te^4+^ vacancy‐ordered double perovskites exhibit strong resistance to pressure, which endows its feasibility in high‐pressure conditions. Moreover, when the studied samples undergo the decompression process, one finds that the emission band can return to its starting state, as described in Figure S14, implying the good structural stability and reversibility of the designed vacancy‐ordered double perovskites. As is known, the STE emission is highly dependent on the electron‐phonon coupling strength and it can be indicated by the Huang‐Rhys factor (*S*). Here, to understand the evolution of the *S* value at high‐pressure, the following calculation formulas are employed [23, 44]:

(10) $$\;E_{Stokes}=2S\hbar w_{\mathrm{LO}}$$

(11) $$\;E_{Stokes}=\frac{hc}{\lambda_{\mathrm{abs}}}\;-\frac{hc}{\lambda_{\mathrm{em}}}$$

where *E_Stokes_* denotes the Stokes shift energy, *ħ* is the reduced Planck constant, *h* is Planck constant, *w*_LO_ is assigned to the longitudinal optical phonon vibration frequency, *c* refers to the speed of light, *λ*_abs_ and *λ*_em_ are the wavelengths of the absorption band maximum and emission band maximum, respectively. Herein, the A_1g_ symmetric longitudinal optical phonons dominate the scattering mechanism by coupling with lattice vibrations [23, 44]. Based on the recorded absorption and emission spectra, the pressure‐dependent *E_Stokes_* values are obtained and depicted in Figure S15a. Thereby, according to the measured Raman spectra as well as the calculated *E_Stokes_* value, the pressure‐associated *S* value is determined and presented in Figure S15b. Obviously, with an increase in pressure, a gradual decrement is seen in the *S* value, i.e., it changes from 11.52 to 6.67 under compression, implying that the pressure can weaken the electron‐phonon coupling strength of the resulting vacancy‐ordered double perovskites. As reported [23, 50], large coupling strength can promote the crossover between the ground state and STE, which will accelerate the annihilation of trapped excitons, resulting in the declined STE emission. Nevertheless, the generation of the STE will be suppressed when the coupling strength is weak, leading to the reduced STE emission. Consequently, through regulating the *S* value to a proper value or a suitable range, enhanced STE emission is expected to be realized in double perovskites. As stated above, the *S* value of the Cs_2_ZrCl_6_:0.008Te^4+^ vacancy‐ordered double perovskites is continuously pressure‐tunable, highlighting the adjustability of the resulting STE emission through high‐pressure engineering, i.e., improved STE emission intensity at high‐pressure.

Aside from the electron‐phonon coupling strength, the changed activation energy barrier for self‐trapping (i.e., *E*_a,trap_) and potential barrier between neighboring STE states (i.e., *E*_p,b_), which is induced by pressure, also have the capacity of impacting the intensity of STE emission [44]. At ambient conditions, carriers with the constant lattice thermal energy overcome the activation barrier (i.e., *E*_a,trap_ or activation energy for detrapping (*E*_a,detrap_)) to perform the exciton state transition, i.e., from the free‐exciton state to STE state or from the STE state to free‐exciton state. Moreover, when pressure elevates, both the *E*_a,trap_ and *E*_p,h_ values are increased gradually. Specifically, the elevated *E*_p,h_, accompanied by an adiabatic potential energy surface that transforms from initially smooth to sharp, will induce STE localization, resulting in the enhanced STE emission intensity at lower pressure. Furthermore, an increase in *E*_p,h_ leads to a reduction in free‐excitons (possessing definite lattice thermal energy) transitioning from the free‐exciton state to STE state, contributing to a continuous decrease in the emission intensity at high‐pressure. Note that, compared with *E*_a,trap_, the response of the *E*_p,b_ to pressure is more sensitive, and thus, the STE emission increases first and then decreases at high‐pressure. The pressure‐induced STE emission evolution mechanism is summarized in Figure S16.

To reveal the impact of pressure on the luminescence properties of the designed compounds in depth, the pressure‐dependent emission band centroid, FWHM and color coordinate of the Cs_2_ZrCl_6_:0.008Te^4+^ vacancy‐ordered double perovskites were estimated and described in Figure 7d–f, respectively. As pressure elevates, the emission band centroid gradually decreases from 582.1 to 532.3 nm (see Figure 7d). Similar phenomenon is also observed in the pressure‐dependent FWHM (see Figure 7c), that is, its value changes from 100.6 to 89.9 nm with the increment of pressure. As is known, the decreased lattice relaxation energy (i.e., *E*_LR_) can contribute to the spectral blue‐shift of the vacancy‐ordered double perovskites, of which the *E*_LR_ value is proportional to FWHM [44, 51], which can be expressed as: $\mathrm{FWHM}\propto \sqrt{2E_{\mathrm{LR}}kT}$. Since the FWHM value weakens at high‐pressure, the *E*_LR_ value will be reduced as pressure rises, leading to the blue‐shift of the STE emission. Besides, the high‐pressure induced bond shortening and octahedral contraction may also account for the blue‐shift of the emission band. Owing to the distinct spectral shift, the Cs_2_ZrCl_6_:0.008Te^4+^ vacancy‐ordered double perovskites exhibit pressure‐triggered polychromatic luminescence, i.e., emitting color changes from yellow to green, with the changed color coordinates, as displayed in Figure 7f. These features endow the Te^4+^‐doped Cs_2_ZrCl_6_ vacancy‐ordered double perovskites with great potential as candidates for optical pressure sensing. For the purpose of studying the manometric properties of the Cs_2_ZrCl_6_:0.008Te^4+^ vacancy‐ordered double perovskites, we first analyzed the relationship between pressure and emission band centroid, which can be described via a third‐order polynomial function, i.e., *λ* = −0.007*p* ^3^ + 0.34*p* ^2^ − 6.36*p* + 581.64 (see Figure 7d). Thus, the pressure‐associated sensitivity, d*λ*/d*p*, is calculated and corresponding results are presented in Figure 7g. As pressure raises, a decrement is seen in the sensitivity and its maximum value is 6.30 nm GPa^−1^. Interestingly, the obtained sensitivity (i.e., d*λ*/d*p*) is not only superior to that of most previously reported luminescent manometers (see Table 1), but also approximately 17.3 times higher than that of ruby, revealing the satisfied manometric properties of the resultant compounds. On the other hand, the pressure‐dependent FWHM also satisfies a third‐order polynomial expression, that is, FWHM = −0.002*p* ^3^ + 0.11*p* ^2^ − 1.89*p* + 101.07, as illustrated in Figure 7e. Thereby, the pressure‐related pressure sensitivity via utilizing FWHM as manometric parameter, i.e., dFWHM/d*p*, is obtained and presented in Figure 7h. It is evident that the maximum sensitivity (i.e., dFWHM/d*p*) is 1.86 nm GPa^−1^, which is comparable with other FWHM‐based optical manometers, as listed in Table 1. Note that, although some recently designed optical manometers, such as Cs_2_Ag_0.6_Na_0.4_InCl_6_:Bi^3+^, Zn_2_GeO_4_:Mn^2+^, etc., exhibit higher sensitivities than the Te^4+^‐doped Cs_2_ZrCl_6_ vacancy‐ordered double perovskites, they all suffer from narrow operating pressure range (i.e., *p* < 10 GPa) and our designed compounds (i.e., up to 20.61 GPa) can efficiently settle this shortage (see Table 1). Additionally, after two compression‐decompression cycles, the STE emission (i.e., fluorescence intensity and emission band position) can still revert to its initial state (see Figure S17), suggesting the splendid stability and reversibility of the Te^4+^‐doped Cs_2_ZrCl_6_ vacancy‐ordered double perovskites, which ensures their applications in high‐pressure conditions.

**TABLE 1:** Comparison of the manometric properties of the Te^4+^‐doped Cs_2_ZrCl_6_ vacancy‐ordered double perovskites with previously designed optical manometers, working in the visible range.

Compounds	Sensitivity (nm GPa^−1^)		Operating range (GPa)	Centroid (nm)	References
	d*λ*/d*p*	dFWHM/d*p*			
Li_4_SrCa(SiO_4_)_2_:Eu^2+^	5.19	1.23	0–10	584.7	[4]
Ba_3_Lu(BO_3_)_3_:Ce^3+^	3.51	—	0–20.1	485	[11]
Sr_2_[Mg_0.9_Li_0.1_Al_4.9_Si_0.1_N_7_]:Eu^2+^	5.07	—	0–10.20	650	[12]
La_3_Mg_2_SbO_9_:Mn^4+^	1.21	0.53	0–9.48	705.99	[13]
NaY_9_(SiO_4_)_6_O_2_:Mn^2+^	7.00	10.13	0.75–7.16	617.2	[15]
Cs_2_Ag_0.6_Na_0.4_InCl_6_:Bi^3+^	112	31.5	0–4	600	[24]
Cs_2_TeCl_6_	3.54	4.82	0–8.05	596.3	[42]
Cs_2_PtCl_6_	7.19	4.95.	0–5.67	677	[51]
Ca_8_Zn(SiO_4_)_4_Cl_2_:Eu^2+^	4.18	1.63	0.46–20.35	500.2	[52]
NaMgBO_3_:Ce^3+^	2.94	—	0.31–17.18	469.3	[53]
Zn_2_GeO_4_:Mn^2+^	21.3	17.0	0–6.76	535.9	[54]
Cs_2_ZrCl_6_:Te^4+^	6.30	1.86	0.13–20.61	582.1	This work

According to Figure 7f, the color coordinates of the designed compounds exhibit a distinct response to pressure. To describe the evolution degree of the color coordinate, we estimated the chromaticity shift, Δ*s*, as defined below [51, 52, 53]:

(12) $$\Delta s\;=\sqrt{{\left(u_{m}^{\prime }-u_{0}^{\prime }\right)}^{2}+{\left(v_{m}^{\prime }-v_{0}^{\prime }\right)}^{2}+{\left(w_{m}^{\prime }-w_{0}^{\prime }\right)}^{2}}\;$$

where *u*′ = 4*x*/(3 − 2*x* + 12*y*), *v*′ = 9*y*/(3 − 2*x* + 12*y*), *w*′ = 1 − *u*′− *v*′, and the color coordinates at initial and studied pressures are presented by 0 and *m*, respectively. With an increase in pressure (i.e., 0.13–20.61 GPa), it is clear that the Δ*s* value increases sharply from 0.07 to 0.22, as displayed in Figure S18, further confirming the significance of the pressure‐induced color coordinate shift, which enables colorimetric pressure sensing. Through analyzing the pressure‐related color coordinates, it is clear that both *x*‐coordinate and *y*‐coordinate aside by a third‐order polynomial expression, i.e., *x* = −1.721 × 10^−5^ *p* ^3^ + 9.064 × 10^−4^ *p* ^2^ − 0.020*p* + 0.468 and *y* = 2.095 × 10^−5^ *p* ^3^ − 9.941 × 10^−4^ *p* ^2^ + 0.013*p* + 0.508, as depicted in Figure S19. For the sake of presenting the pressure sensing capacity of the resulting vacancy‐ordered double perovskites, the relative pressure sensitivity (i.e., *S_R_*) is determined, as described below [51, 52, 53]:

(13) $$\;S_{R}=\left|\frac{1}{C}\frac{dC}{dp}\right|\times 100\%$$

where the color coordinate is labelled by *C* and the relative pressure sensitivities estimated from *x*‐coordinate and *y*‐coordinate are described by *S_R_* (*x*) and *S_R_* (*y*), respectively. As demonstrated, the maximum *S_R_* (*x*) and *S_R_* (*y*) values of the Cs_2_ZrCl_6_:0.008Te^4+^ vacancy‐ordered double perovskites are 4.25% and 2.50% GPa^−1^ (see Figure 7i), respectively, demonstrating that the resulting compounds can be used as colorimetric pressure sensing. These results manifest that Te^4+^‐doped Cs_2_ZrCl_6_ vacancy‐ordered double perovskites, with good pressure sensitivities, are promising pressure‐sensitive luminescent materials for multi‐parameter optical manometry, i.e., using the emission band centroid and color coordinate as the pressure indicators.

## 3 Conclusions

In summary, a facile rapid room‐temperature precipitation method is proposed to synthesize the Tb^4+^‐doped Cs_2_ZrCl_6_ vacancy‐ordered double perovskites. Excited by 413 nm, the designed compounds can emit intense broad emission band at 580 nm, pertaining to the STE emission, of which the optimal doping content is *x* = 0.008 and the involved concentration quenching mechanism is electric dipole‐dipole interaction. When the resulting vacancy‐ordered double perovskites are exposed to a low‐temperature environment, their luminescence properties (i.e., emission band centroid, bandwidth and lifetime) are modulated, unlocking their potential for temperature measurement in the cryogenic range. Specifically, when the emission band and lifetime are adopted as the temperature indictors, the *S_R_* (*T*) values of the resulting vacancy‐ordered double perovskites are determined to be 0.48% and 0.81% K^−1^, respectively. In situ high‐pressure absorption spectra reveal the simultaneous reduction of the optical band gap and piezochromism in the developed compounds. Moreover, the designed vacancy‐ordered double perovskites possess good structural stability and reversibility within the applied pressure range of 0.13–22.03 GPa, as verified by the in situ high‐pressure Raman spectra and XRD patterns, ensuring their operating in high‐pressure conditions. With an increase in pressure, an evident spectral blue‐shift, i.e., from 582.1 to 532.2 nm, happens in the final products, contributing to the pressure‐triggered polychromic luminescence. Furthermore, the bandwidth of the STE emission is also dependent on the pressure, namely, the FWHM declines from 100.6 to 89.9 nm as pressure rises. Notably, via utilizing the emission band centroid and FWHM as manometric parameters, the maximum pressure sensitivities of the studied samples are 6.30 and 1.86 nm GPa^−1^, respectively, of which the corresponding operating range up to 20.61 GPa. Additionally, based on the pressure‐dependent color coordinate, the developed double perovskites can be used as colorimetric pressure sensing and the maximum *S_R_* value is 4.25% GPa^−1^. These results clarify that luminescence behaviors the Te^4+^‐doped Cs_2_ZrCl_6_ vacancy‐ordered double perovskites can be efficiently regulated via extreme conditions, endowing their promising applications in advanced optical manometry and thermometry.

## 4 Experimental Section

### 4.1 Preparation of the Te^4+^‐Doped Cs_2_ZrCl_6_ Vacancy‐Ordered Double Perovskites

Via using a room‐temperature preparation method, series of the Cs_2_ZrCl_6_:*x*Te^4+^ (0.004 ≤ *x* ≤ 0.012) vacancy‐ordered double perovskites were synthesized. The high‐purity powders of CsCl, ZrCl_4_ and TeCl_4_, in which their purities were 99.95%, 99.5% and 99.9%, respectively, were bought from Aladdin Company and employed as the raw materials. According to the designed compounds, the proper amounts of these above powders were weighted and kept in beaker. After that, 3 mL of HCl was added and stirred for 15 min to form the precipitation. Then, the centrifugation process was performed and washed with ethanol for three times. Subsequently, it dried at 80°C in an oven for 2 h. Finally, the obtained products were grounded and collected for further characterization. Herein, the reaction yield was found to be around 86.5%. Furthermore, to explore the impact of the reaction time and temperature on the luminescence behaviors of the studied samples, different products were also prepared, i.e., (i) for the reaction temperature related samples, the reaction time was fixed at 15 min, whereas the reaction temperature changed from 30°C to 90°C and (ii) for the reaction time associated compounds, the reaction temperature was fixed at room‐temperature, while the reaction time altered from 15 to 120 min.

### 4.2 Sample Characterizations

The phase and elemental compositions of the designed double perovskites were checked through an X‐ray diffractometer (Bruker D8 Advance) and X‐ray photoelectron spectrometer (PHI 5000 VersaProbe III), respectively. By means of a field‐emission SEM (Hitachi SU3500) and TEM (JEM‐2100F, JEOL), the particle size and shape of the prepared samples were examined. The room‐temperature excitation and emission spectra as well as the decay curves were measured by a fluorescence spectrometer (Edinburgh FS5). The temperature‐dependent emission spectra, decay curves and quantum efficiency of the studied samples were recorded using a fluorescence spectrometer (Edinburgh FSL1000). The excitation power density related emission spectra were measured by a fluorescence spectrometer (Ocean Optics Maya2000Pro), in which a power tunable LED was attached as the excitation source.

In situ high‐pressure measurements were carried out utilizing a symmetric DAC, of which the gasket (i.e., T301 steel) was pre‐indented to 40 µm and drilled a hole of 150 µm. The resulting compound and ruby were kept in the hole for further measurement, where the generated pressure in DAC was calibrated using the ruby fluorescence. To detect the pressure‐dependent emission spectra, a 365 nm laser diode was used. The in situ high‐pressure Raman spectra was tested by a micro‐Raman spectrometer (Mono Vista CRS+500) with 532 nm laser as excitation source. The in situ XRD patterns at high‐pressure were conducted at BL17U1 beamline of Shanghai Synchrotron Radiation Facility (SSRF). The equation of state of Au was used to measure the pressure in the high‐pressure chamber. The obtained one‐dimensional angle dispersive X‐ray diffraction patterns were integrated into two‐dimensional data with Dioptas software.

## Conflicts of Interest

The authors declare no conflicts of interest.

## Supporting information




**Supporting File**: advs74378‐sup‐0001‐SuppMat.docx

## Data Availability

The data that support the findings of this study are available from the corresponding author upon reasonable request.
