# Supplementary material for: Rapid Room‐Temperature Synthesis of Te4+‐Doped Cs2ZrCl6 Vacancy‐Ordered Double Perovskites With Tunable Luminescence Triggered by Extreme Conditions for Advanced Optical Thermometry and Manometry
Source: Adv Sci (Weinh). 2026 Feb 23;13(23):e23940. doi: 10.1002/advs.202523940 (PMC13104067; doi:10.1002/advs.202523940)
Supplement: Supplementary file 1 — Supporting File: advs74378‐sup‐0001‐SuppMat.docx [file ADVS-13-e23940-s001.docx]

**Rapid Room-Temperature Synthesis of Te^4+^-Doped Cs_2_ZrCl_6_ Vacancy-Ordered Double Perovskites with Tunable Luminescence Triggered by Extreme Conditions for Advanced Optical Thermometry and Manometry**

*Zhiyu Pei, Yeshan Wu, Shuailing Ma^*^, Tian Cui, Laihui Luo^*^, Peng Du^*^*

P. Du, Z. Liu, Z. Pei, S. Ma, T. Cui

School of Physical Science and Technology, Ningbo University, 315211 Ningbo, Zhejiang, China

^*^E-mail: mashuailing@nbu.edu.cn (S. Ma); luolaihui@nbu.edu.cn (L. Luo); dupeng@nbu.edu.cn (P. Du)

**Table S1** Lattice parameters of the Cs_2_ZrCl_6_:0.008Te^4+^ and Cs_2_ZrCl_6_:0.012Te^4+^ vacancy-ordered double perovskites.

| Parameter | Compounds | |
| --- | --- | --- |
|  | Cs_2_ZrCl_6_:0.008Te^4+^ | Cs_2_ZrCl_6_:0.012Te^4+^ |
| *a* = *b* = *c* (Å) | 10.437741 | 10.43848 |
| Volume (Å^3^) | 1137.155 | 1137.396 |
| *R_p_* | 15.98% | 14.93% |
| *R_wp_* | 23.49% | 20.97% |
| χ^2^ | 1.33 | 1.12 |


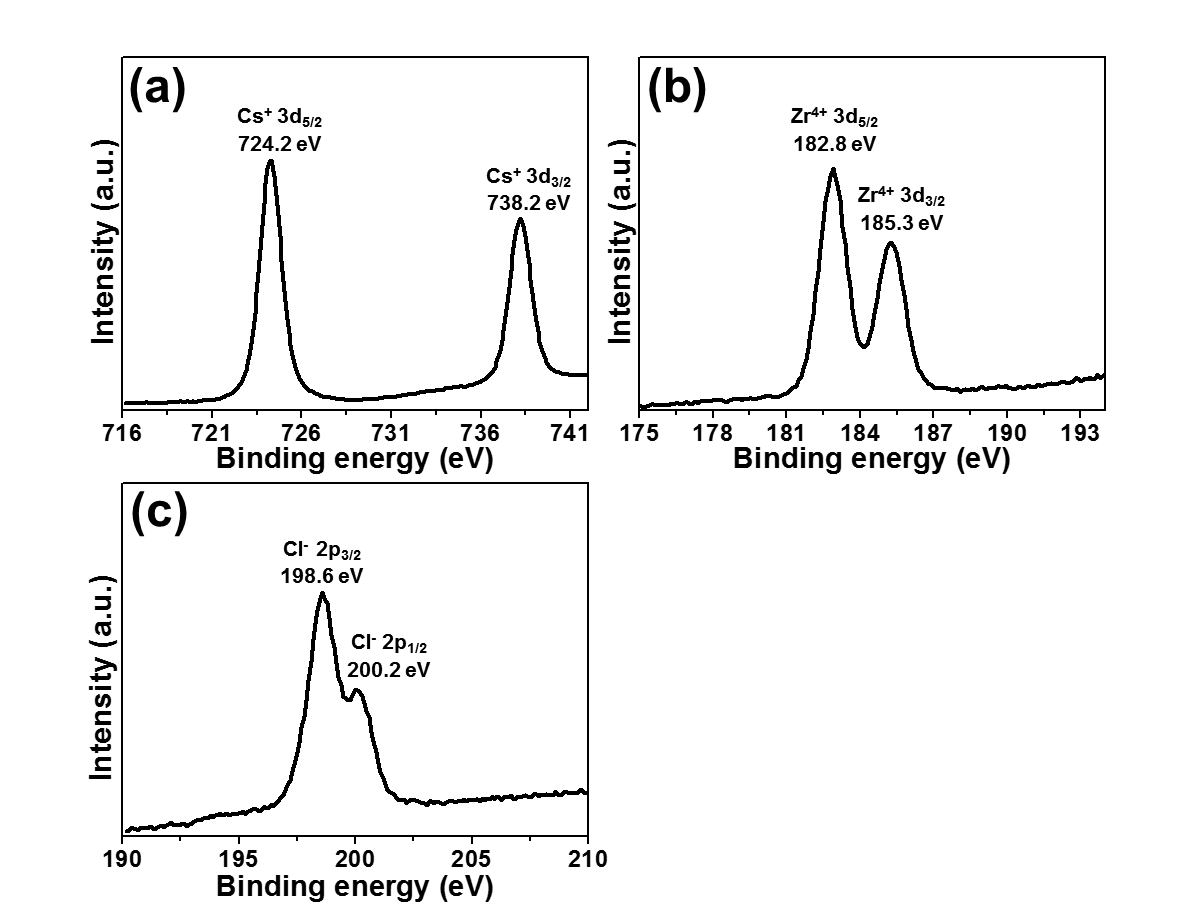


**Figure S1** High-resolution XPS spectra of (a) Cs^+^ 3d, (b) Zr^4+^ 3d and (c) Cl^-^ 2p in the Cs_2_ZrCl_6_:0.008Te^4+^ vacancy-ordered double perovskites.


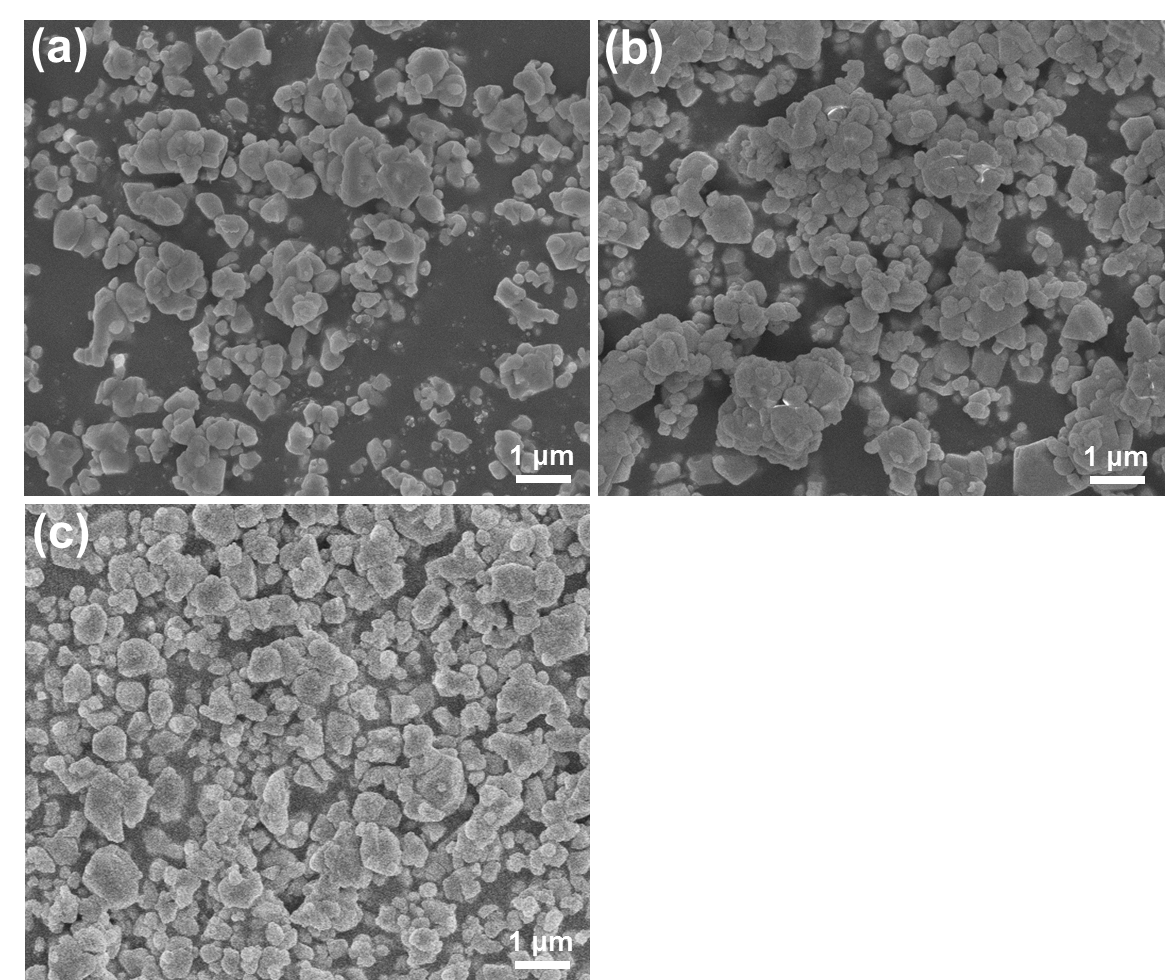


**Figure S2** SEM images of the Cs_2_ZrCl_6_:*x*Te^4+^ vacancy-ordered double perovskites with the dopant content of (a) *x* = 0.004, (b) *x* = 0.006 and (c) *x* = 0.010.


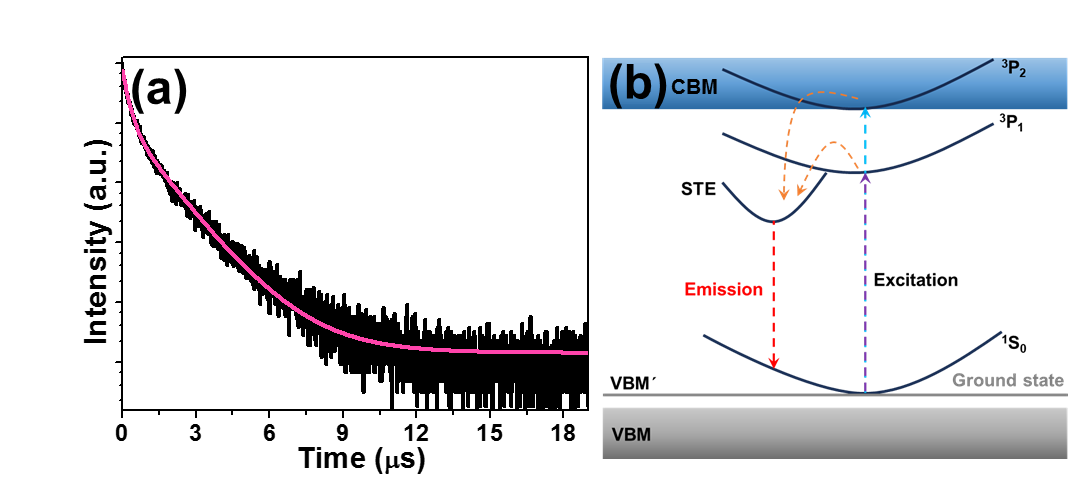


**Figure S3** (a) Decay curve of the Cs_2_ZrCl_6_:0.008Te^4+^ vacancy-ordered double perovskites. (b) Energy level diagrams of the host and Te^4+^ as well as the possible luminescence process. CBM: conduction band maximum; VBM: Valence band minimum.

Figure S3(a) shows the decay curve of the Cs_2_ZrCl_6_:0.008Te^4+^ vacancy-ordered double perovskites, of which the monitoring and excitation wavelengths are 580 and 413 nm, respectively. Herein, the decay curve can be fitted by a double exponential expression, as described below:

$I\left( t \right)=A_{1}\exp\left( {-t}/{\tau_{1}} \right)+A_{2}\exp\left( {-t}/{\tau_{2}} \right)$ (S1)

where *I*(t) refers to the fluorescence intensity at time *t*, *A*_1_ and *A*_2_ are constants, *τ*_1_ and *τ*_2_ are decay time. Accordingly, the average lifetime (*τ*_avg_) can be obtained via using the following function:

$\tau_{avg}=\frac{A_{1}\tau_{1}^{2}+A_{2}\tau_{2}^{2}}{A_{1}\tau_{1}+A_{2}\tau_{2}}$ (S2)

Thus, the lifetime of the emission at 580 nm in the Cs_2_ZrCl_6_:0.008Te^4+^ vacancy-ordered double perovskites is found to be 1.54 μs.


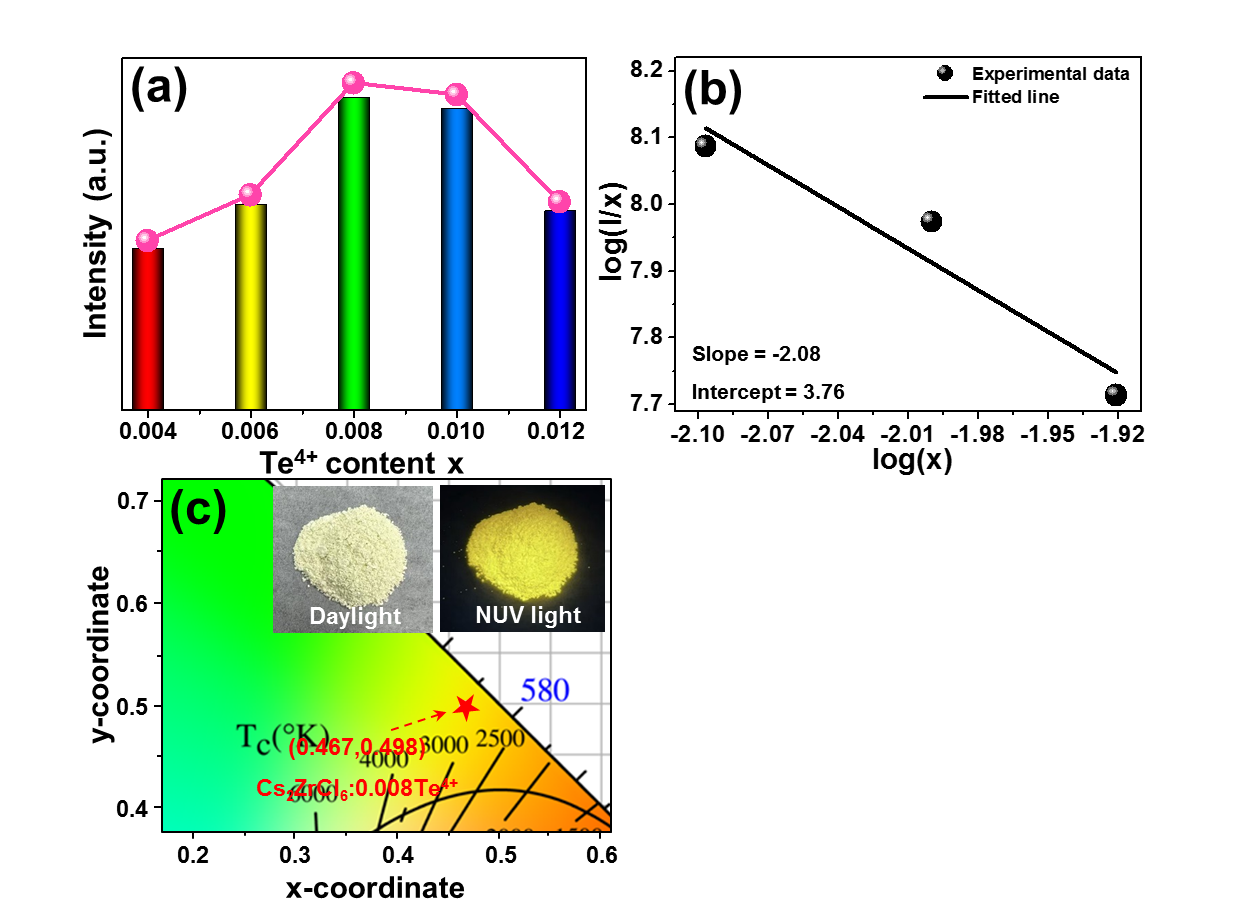


**Figure S4** (a) Emission intensity and (b) plot of log(I/*x*) *vs*. log(*x*) for the Cs_2_ZrCl_6_:*x*Te^4+^ vacancy-ordered double perovskites. (c) CIE chromaticity diagram of the Cs_2_ZrCl_6_:0.008Te^4+^ vacancy-ordered double perovskites as well as its optical images excited by different lights.

To explore the concentration quenching mechanism in the studied samples, the critical distance, *R_c_*, is calculated via the following function:^[1]^

$R_{c}=2\left( \frac{3V}{4\pi x_{c}Z} \right)^{1/3}$ (S3)

where *V* refers to the unit cell volume, *x*_c_ is the critical dopant content and *Z* refers to the amount of cations in the unit cell. Here, the values of *x_c_*, *V* and *Z* are 0.008, 1137.155 Å^3^ and 4, respectively, and thus, the *R_c_* value in the Cs_2_ZrCl_6_ host lattices is calculated to be 40.8 Å, which is larger than 5 Å, suggesting that the concentration quenching mechanism is contributed by the electric dipolar interaction. To get deeper insight into the involved concentration quenching mechanism, the following formula is adopted to analyze the relation between fluorescence intensity (*i.e*., I) and dopant content (*i.e*., *x*):^[2]^

$\log\left( \frac{I}{x} \right)=A-\frac{\theta}{3}log\left( x \right)$ (S4)

where the value of *θ* can be 6, 8 and 10 corresponds to the electric dipole-dipole, dipole-quadrupole and quadrupole-quadrupole interactions, respectively. Through linearly fitting the experimental data (Figure S4(b)), one knows that slope (-*θ*/3) of the fitted line is -2.08, and thus, the *θ* value is determined to be 6.24, which is close to 6, suggesting that the electric dipole-dipole interaction contributes to the concentration quenching in the Te^4+^-doped Cs_2_ZrCl_6_ vacancy-ordered double perovskites.

**References**

1. G. Blasse, *Phys. Lett. A* **1968**, *28*, 444-445.

2. D. L. Dexter, *J. Chem. Phys*. **1953**, *21*, 836-850.


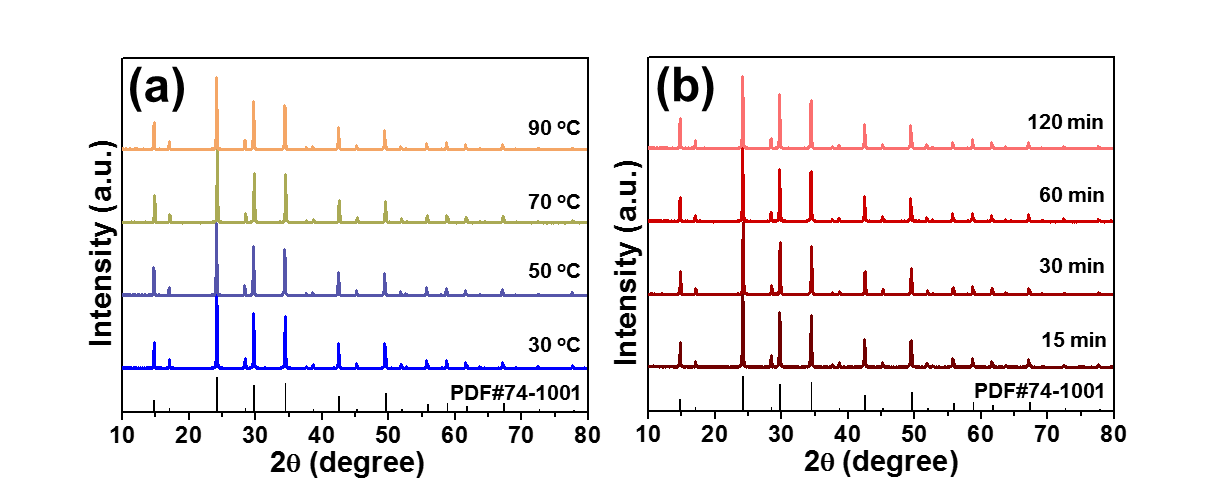


**Figure S5** XRD patterns of the Cs_2_ZrCl_6_:0.008Te^4+^ vacancy-ordered double perovskites prepared by different reaction (a) temperature and (b) time.


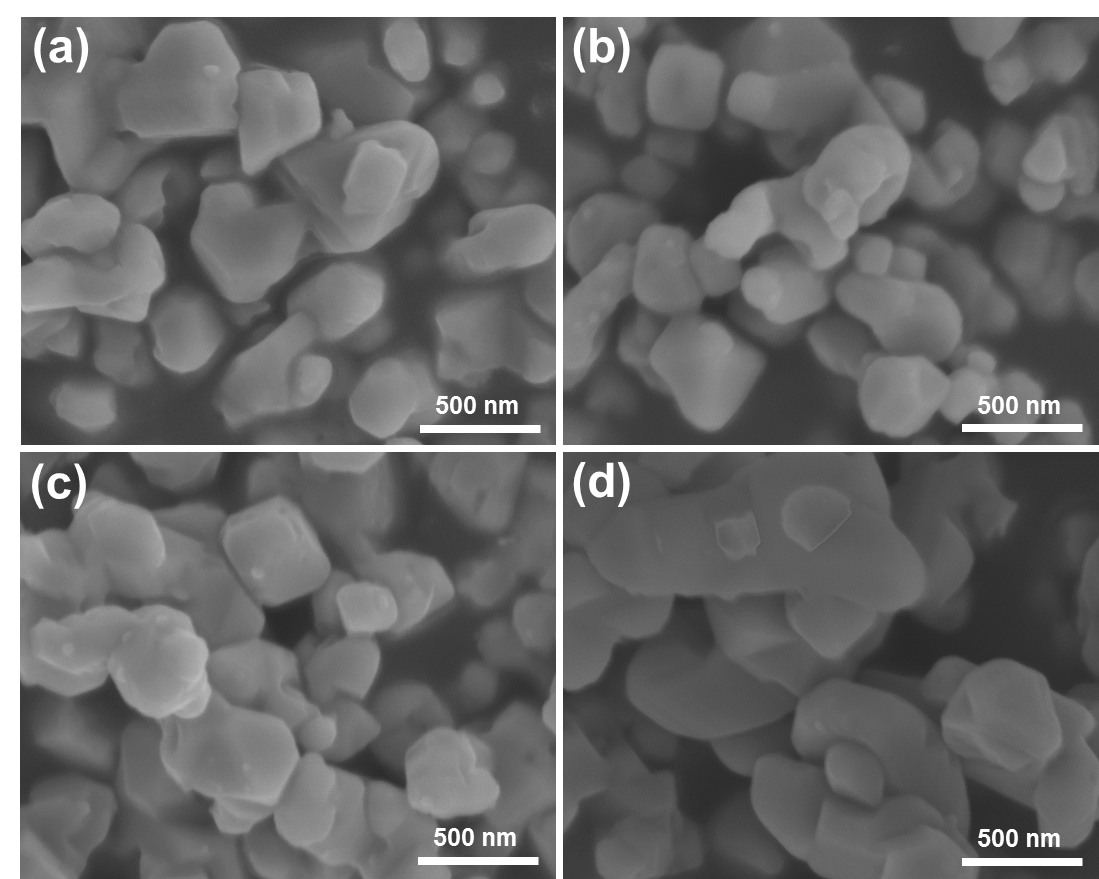


**Figure S6** SEM images of the Cs_2_ZrCl_6_:0.008Te^4+^ vacancy-ordered double perovskites prepared by different reaction temperature of (a) 30 °C, (b) 50 °C, (c) 70 °C and (d) 90°C, in which the reaction time is fixed at 15 min.


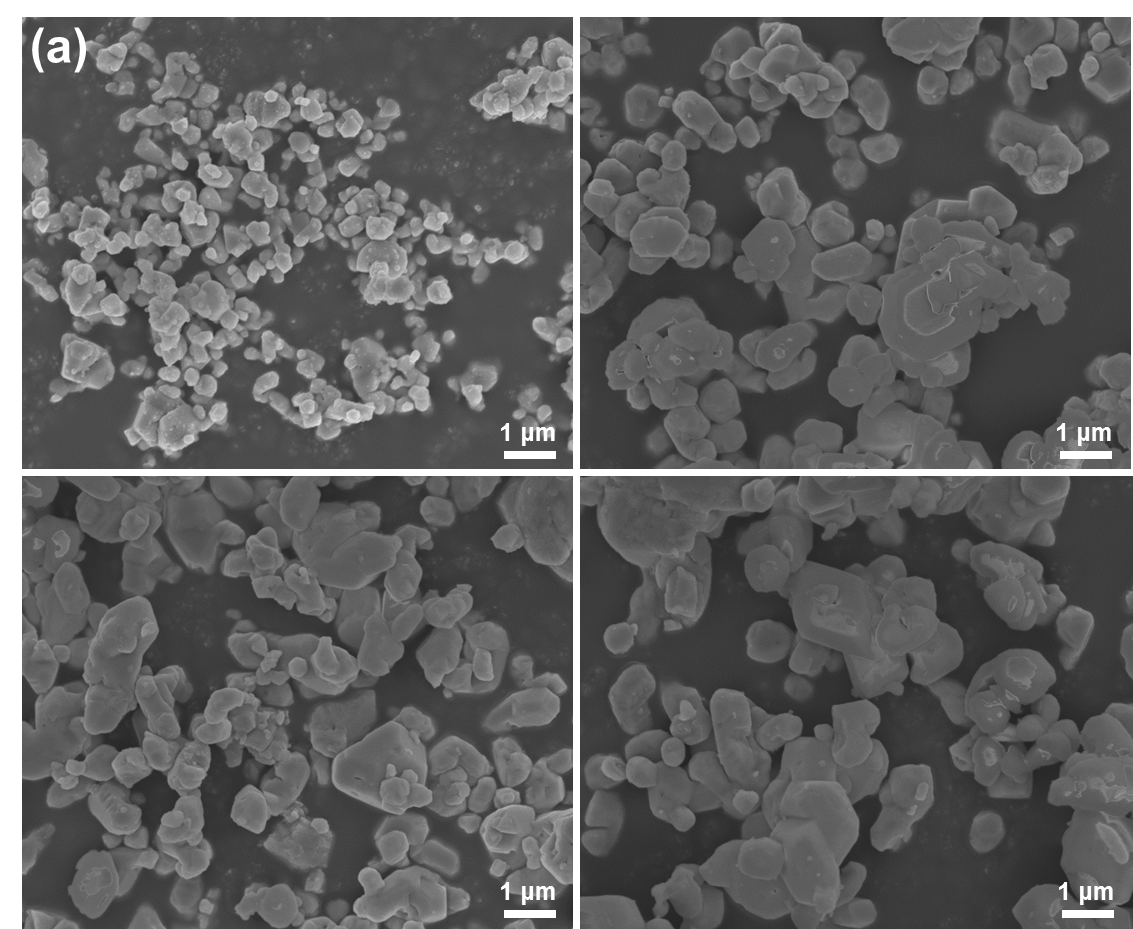


**Figure S7** SEM images of the Cs_2_ZrCl_6_:0.008Te^4+^ vacancy-ordered double perovskites prepared by different reaction time of (a) 15 min, (b) 30 min, (c) 60 min and (d) 120 min, in which the reaction temperature is room-temperature.


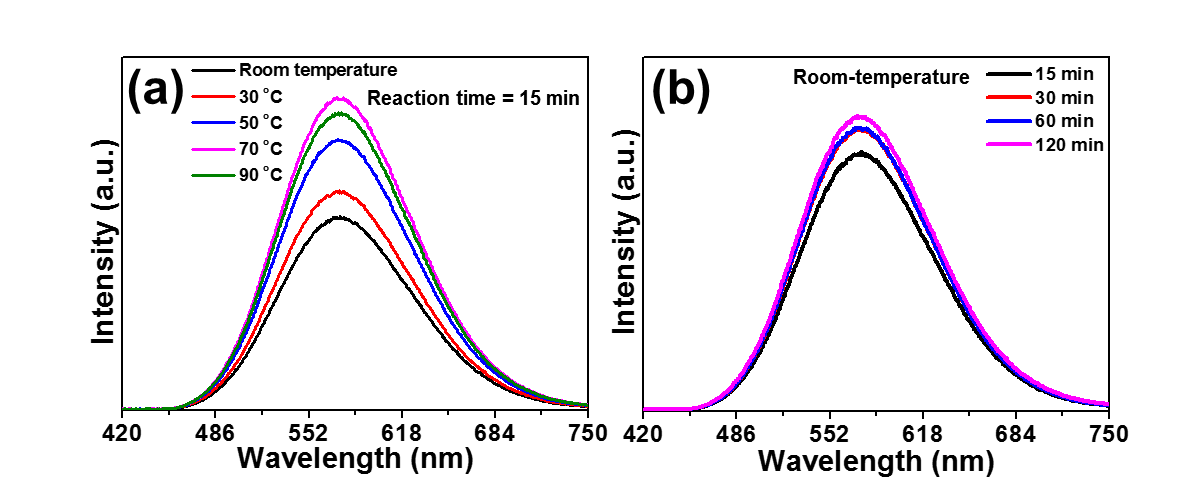


**Figure S8** Emission spectra of the Cs_2_ZrCl_6_:0.008Te^4+^ vacancy-ordered double perovskites prepared by different reaction (a) temperature and (b) time.


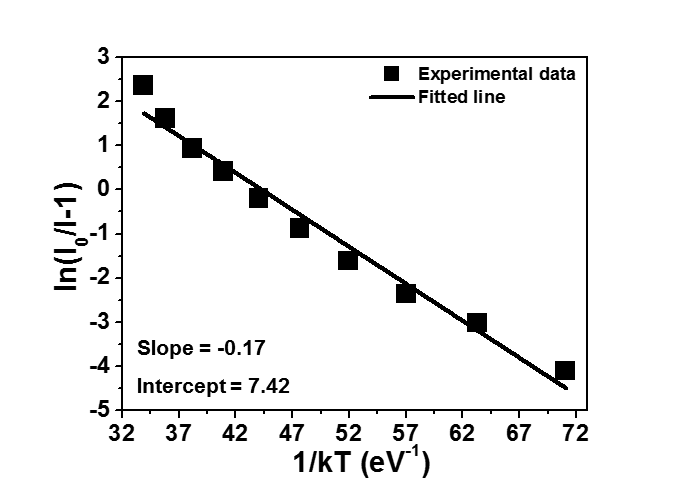


**Figure S9** Plot of ln(I_0_/I-1) *vs*. 1/*kT* for the Cs_2_ZrCl_6_:0.008Te^4+^ vacancy-ordered double perovskites.


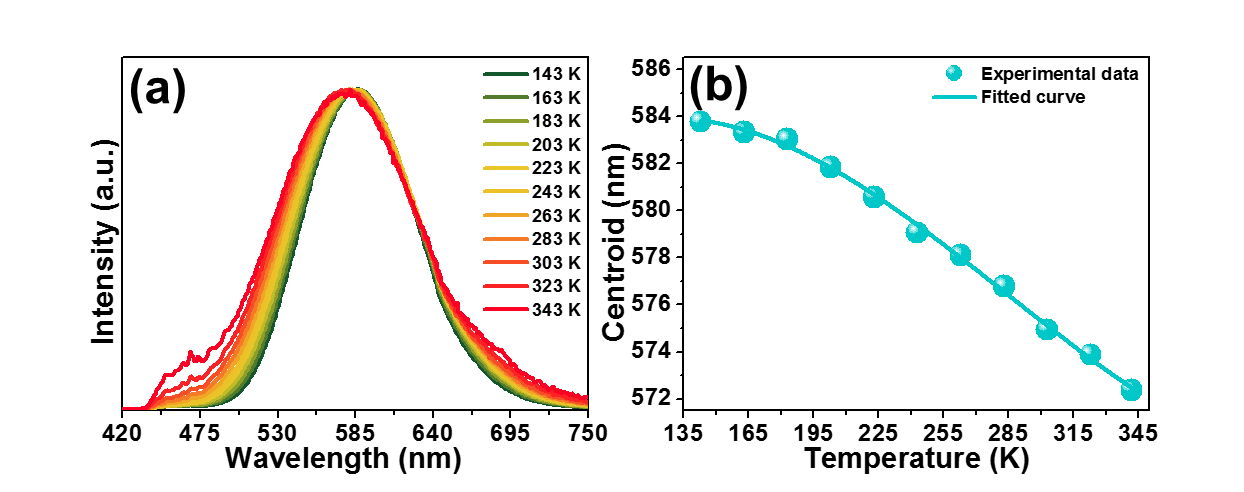


**Figure 10** Temperature-dependent (a) emission spectra and (b) emission band centroid of the Cs_2_ZrCl_6_:0.008Te^4+^ vacancy-ordered double perovskites.


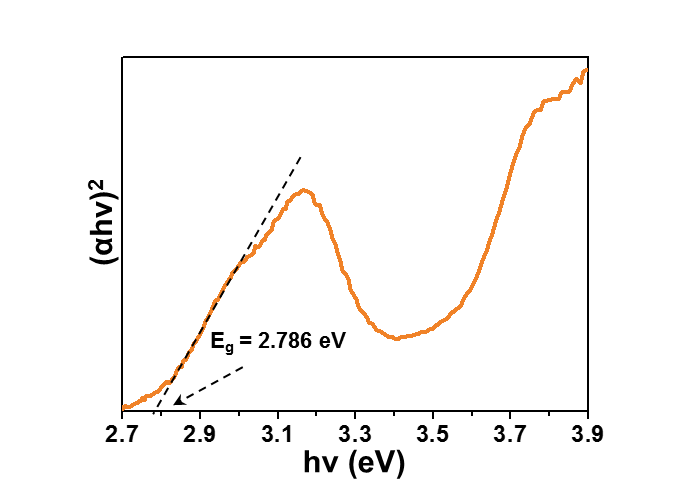


**Figure S11** Plot of (*αhv*)^2^ *vs*. *hv* for the the Cs_2_ZrCl_6_:0.008Te^4+^ vacancy-ordered double perovskites recorded at 0.13 GPa during the decompression process.


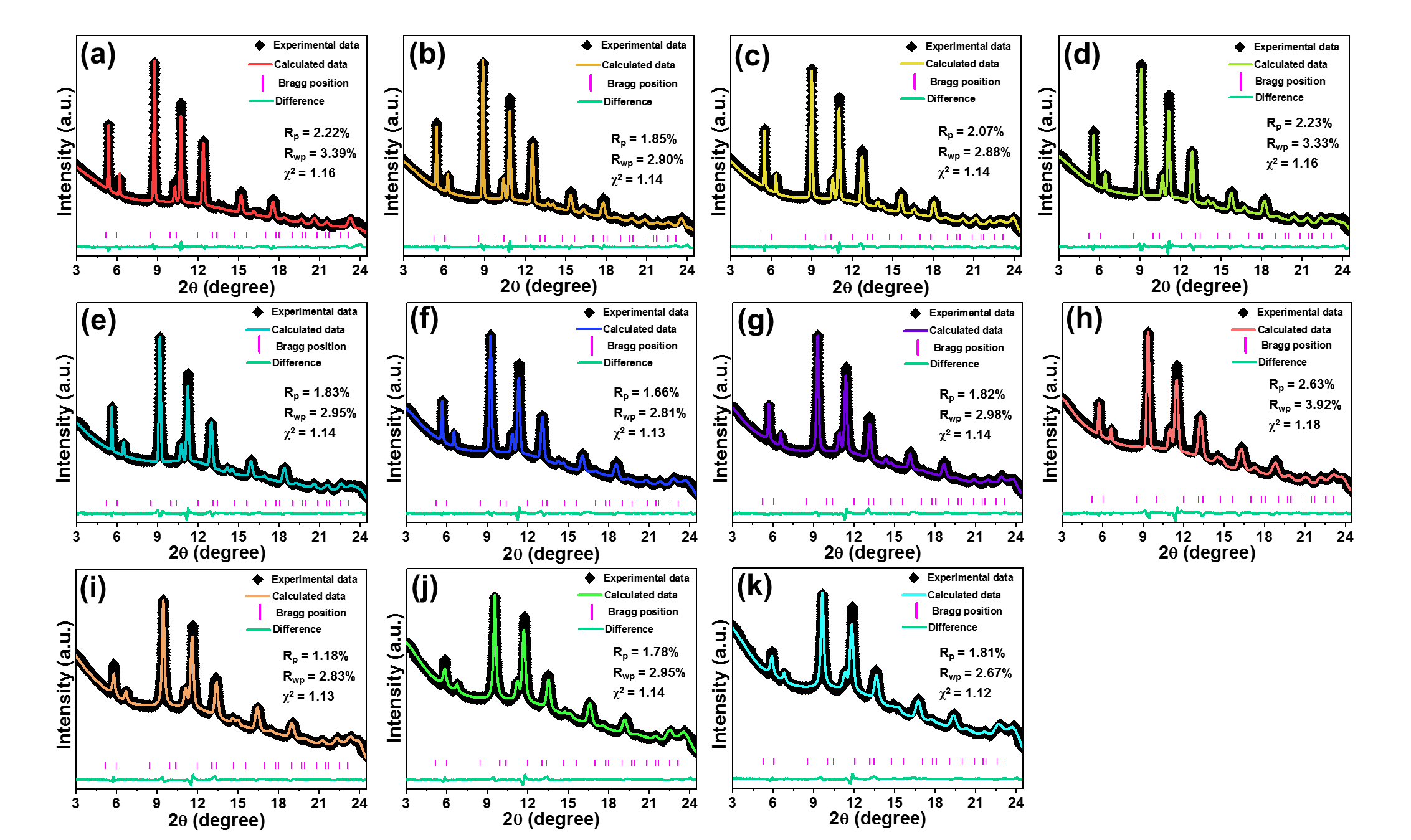


**Figure S12** Rietveld XRD refinements of the Cs_2_ZrCl_6_:0.008Te^4+^ vacancy-ordered double perovskites recorded at (a) *p* = 1.43, (b) *p* = 2.83, (c) *p* = 4.43, (d) *p* = 5.93, (e) *p* = 7.33, (f) *p* = 9.33, (g) *p* = 10.73, (h) *p* = 11.93, (i) *p* = 13.33, (j) *p* = 16.03 and (k) *p* = 20.13 GPa.


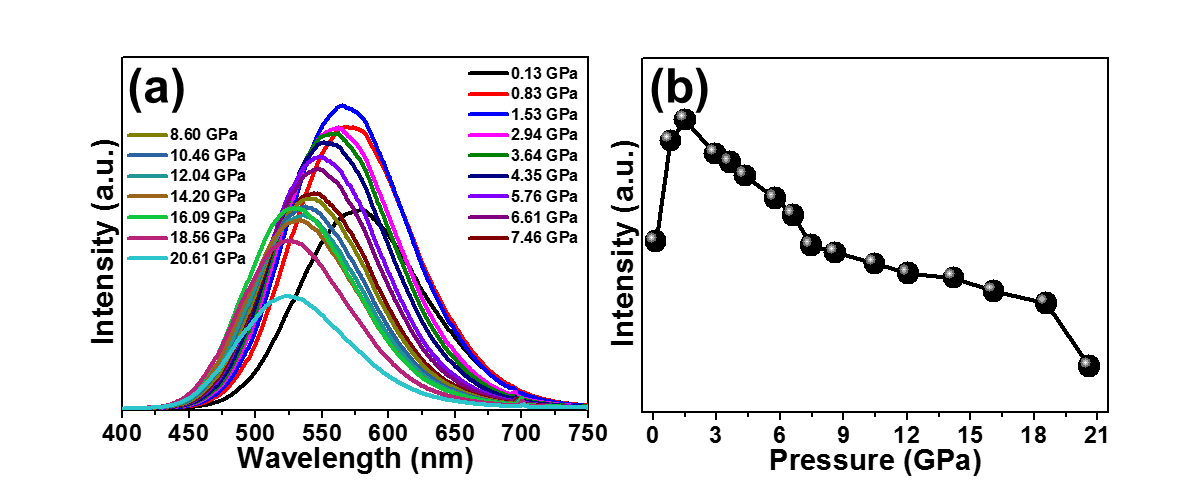


**Figure S13** (a) Emission spectra and (b) Fluorescence intensity of the Cs_2_ZrCl_6_:0.008Te^4+^ vacancy-ordered double perovskites as a function of pressure.


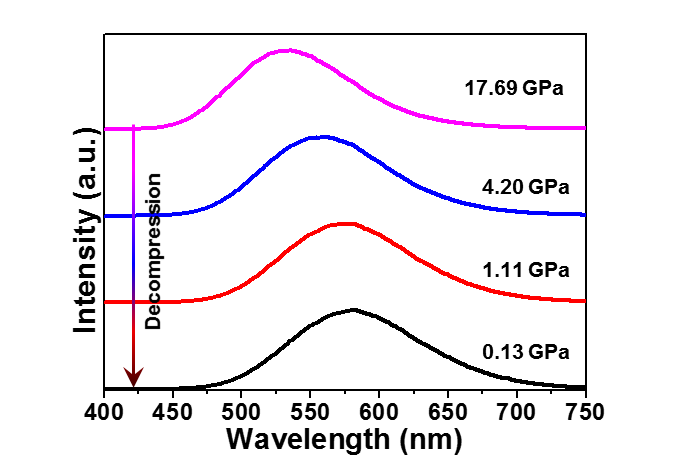


**Figure S14** Emission spectra of the Cs_2_ZrCl_6_:0.008Te^4+^ vacancy-ordered double perovskites during the decompression process.


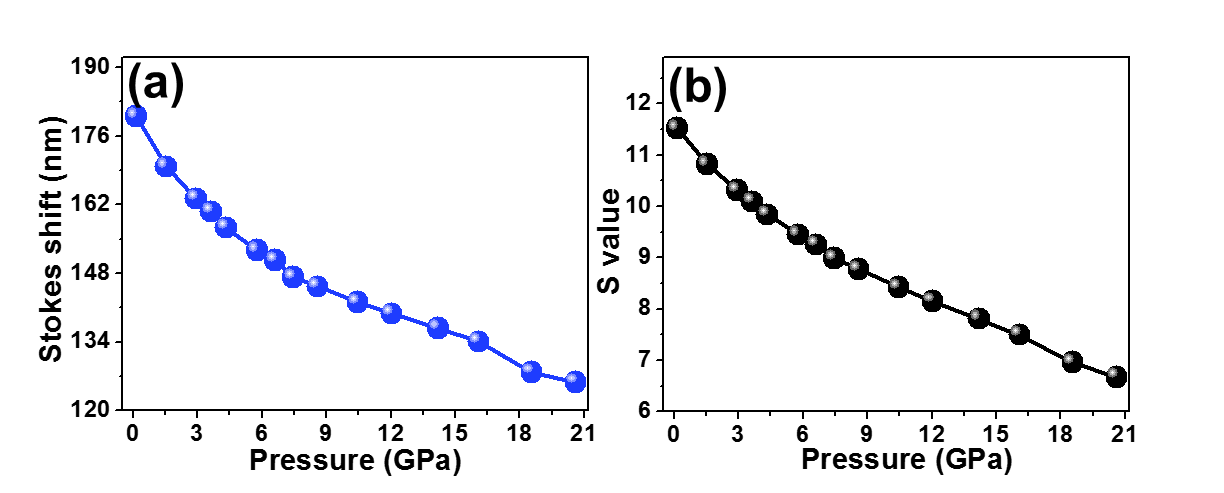


**Figure S15** Pressure-related (a) Strokes shift and (b) *S* value of the Cs_2_ZrCl_6_:0.008Te^4+^ vacancy-ordered double perovskites during the compression process.


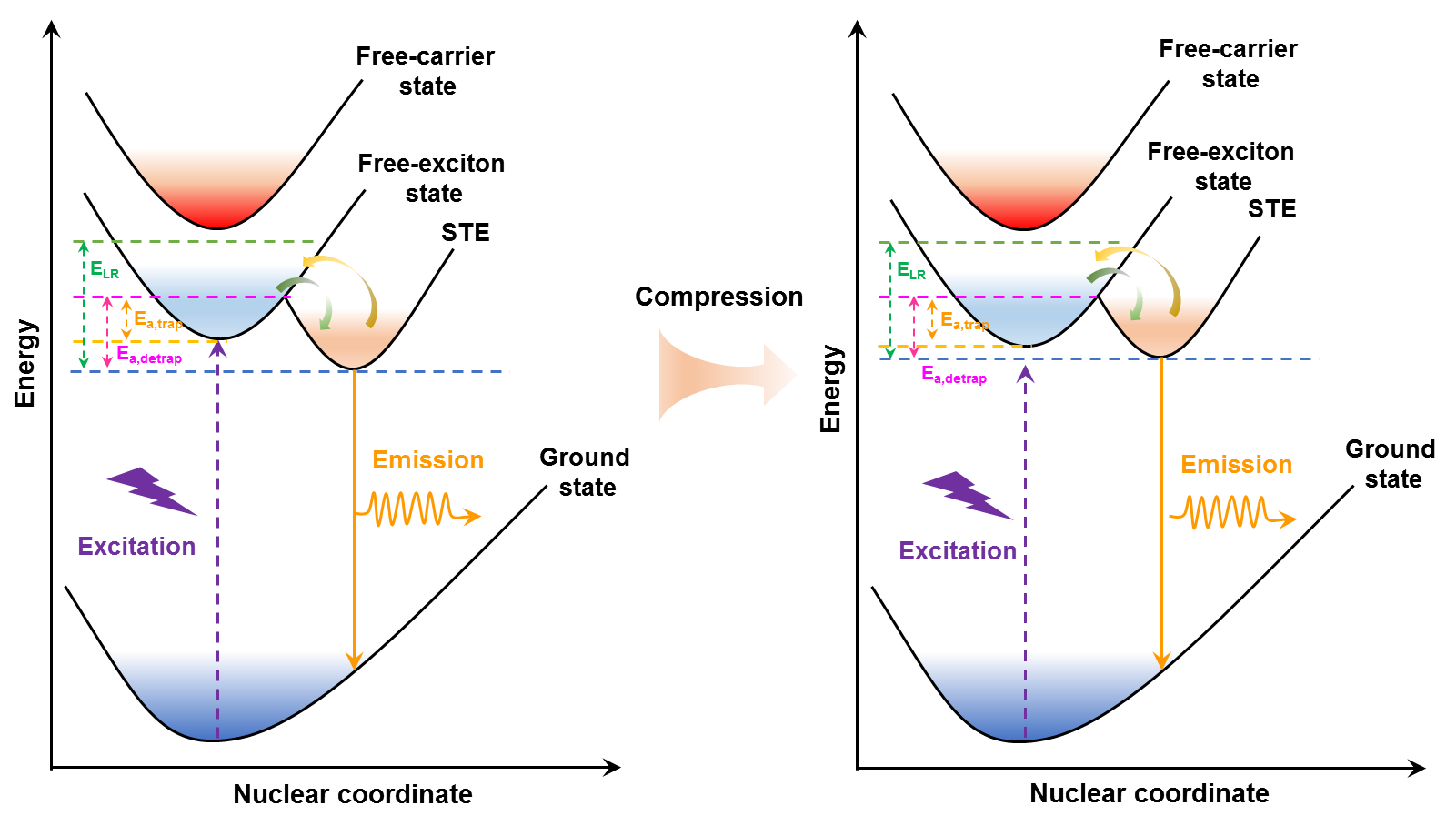


**Figure S16** Schematic illustration of the luminescence evolution at high-pressure conditions.


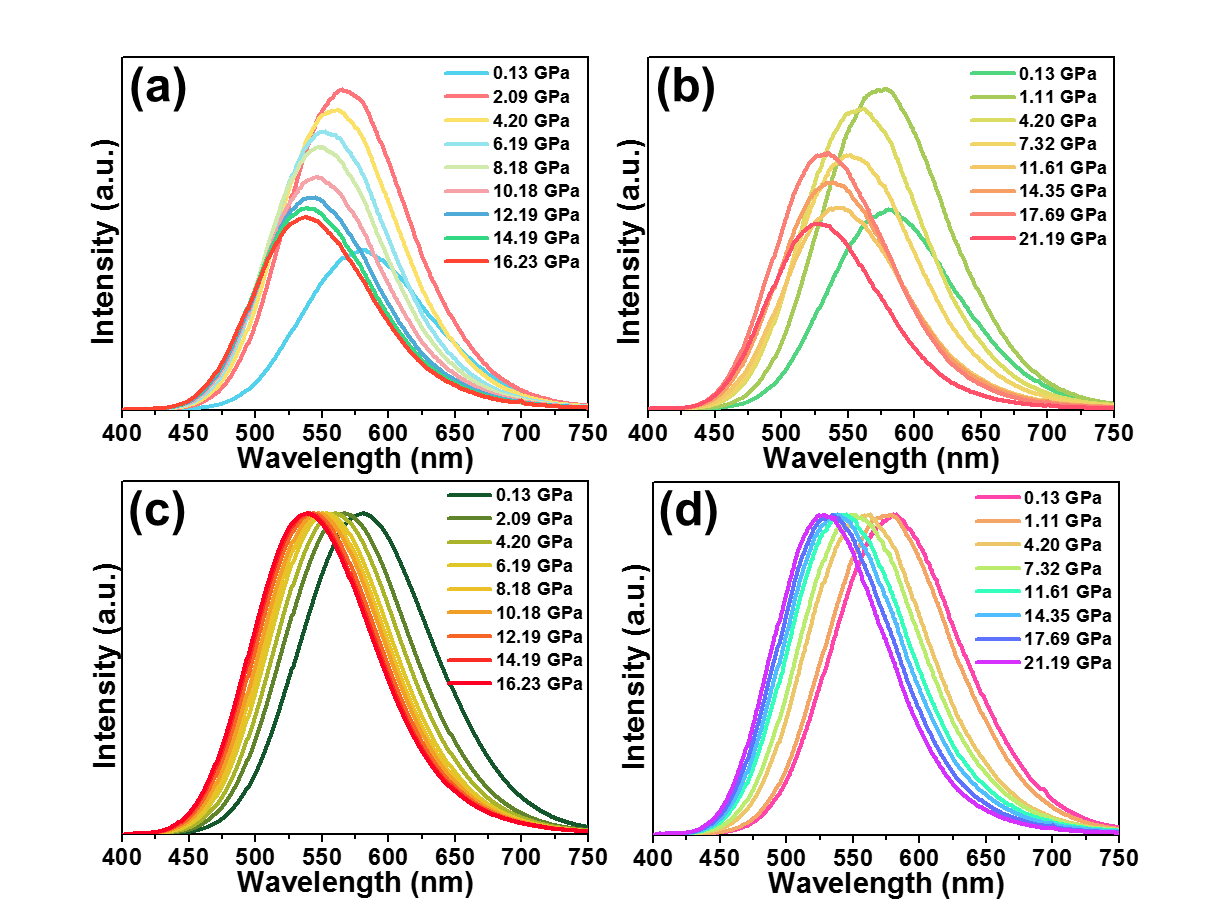


**Figure S17** Emission spectra of Cs_2_ZrCl_6_:0.008Te^4+^ vacancy-ordered double perovskites in the pressure range of (a) 0.13-16.23 GPa and (b) 0.13-21.19 GPa. Normalized emission spectra of Cs_2_ZrCl_6_:0.008Te^4+^ vacancy-ordered double perovskites in the pressure range of (c) 0.13-16.23 GPa and (d) 0.13-21.19 GPa.


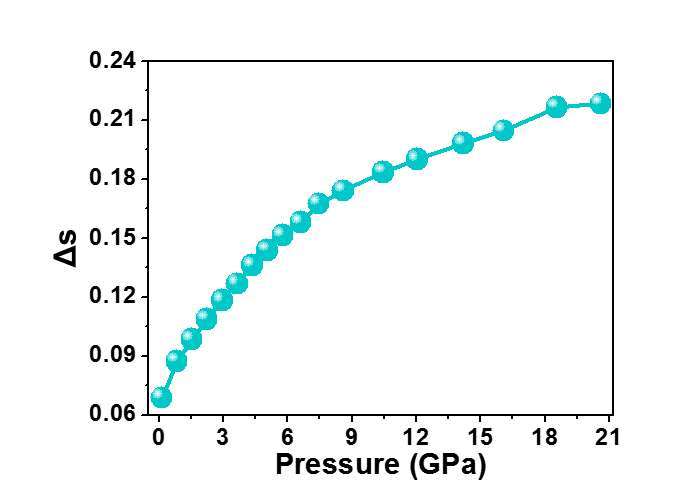


**Figure S18** Δs value of the Cs_2_ZrCl_6_:0.008Te^4+^ vacancy-ordered double perovskites as function of pressure.


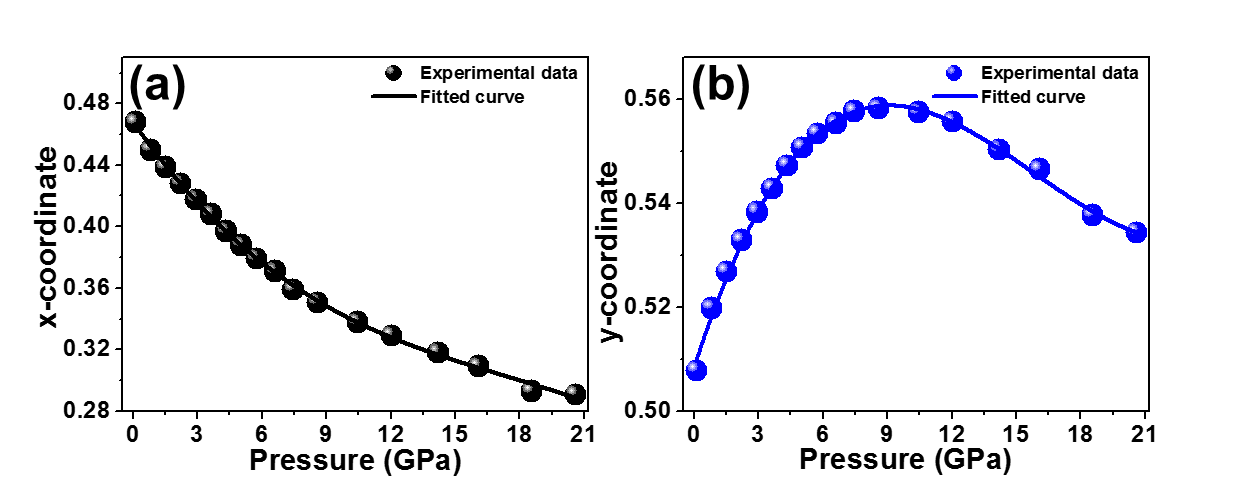


**Figure S19** Pressure-dependent (a) *x*-coordinate and (b) y-coordinate of the Cs_2_ZrCl_6_:0.008Te^4+^ vacancy-ordered double perovskites.
